# The sex of specific neurons controls female body growth in *Drosophila*

**DOI:** 10.1371/journal.pbio.2002252

**Published:** 2017-10-04

**Authors:** Annick Sawala, Alex P. Gould

**Affiliations:** The Francis Crick Institute, London, United Kingdom; ICM Institute, France

## Abstract

Sexual dimorphisms in body size are widespread throughout the animal kingdom but their underlying mechanisms are not well characterized. Most models for how sex chromosome genes specify size dimorphism have emphasized the importance of gonadal hormones and cell-autonomous influences in mammals versus strictly cell-autonomous mechanisms in *Drosophila melanogaster*. Here, we use tissue-specific genetics to investigate how sexual size dimorphism (SSD) is established in *Drosophila*. We find that the larger body size characteristic of *Drosophila* females is established very early in larval development via an increase in the growth rate per unit of body mass. We demonstrate that the female sex determination gene, *Sex-lethal* (*Sxl*), functions in central nervous system (CNS) neurons as part of a relay that specifies the early sex-specific growth trajectories of larval but not imaginal tissues. Neuronal Sxl acts additively in 2 neuronal subpopulations, one of which corresponds to 7 median neurosecretory cells: the insulin-producing cells (IPCs). Surprisingly, however, male-female differences in the production of insulin-like peptides (Ilps) from the IPCs do not appear to be involved in establishing SSD in early larvae, although they may play a later role. These findings support a relay model in which Sxl in neurons and Sxl in local tissues act together to specify the female-specific growth of the larval body. They also reveal that, even though the sex determination pathways in *Drosophila* and mammals are different, they both modulate body growth via a combination of tissue-autonomous and nonautonomous inputs.

## Author summary

Males and females differ in size in many animal species. This reflects differences in body growth during development that are under the control of sex chromosome genes. In mammals, sex chromosome genes act in the gonads to instruct them to produce secreted hormones that regulate male-female differences in body growth. In contrast, in the fruit fly *Drosophila melanogaster*, it has long been thought that the larger body size of females does not involve secreted hormones but instead depends upon the local action of the sex determination gene *Sex-lethal* in every cell of the body. We now show that *Drosophila* females grow faster than males during the early phase of larval development. Surprisingly, the early increased growth of the female body depends upon *Sex-lethal* expression in 2 groups of neurons in the brain, one of which is known to be neurosecretory. These findings indicate that *Sex-lethal* not only functions locally to regulate the growth of the cells in which it is expressed but that it also acts more globally via secreted factors that remotely control the growth of many different tissues. *Drosophila* and mammalian sex chromosome genes, although different, may therefore regulate body growth in a more similar way than was previously thought.

## Introduction

The sex of an organism has profound effects on its morphology, physiology, and behaviour. It also influences the risk of developing diseases of growth and metabolism such as obesity, metabolic syndrome, cardiovascular disease, and cancer [1,2]. One important feature of sexual dimorphism is the difference in body size between males and females, called sexual size dimorphism (SSD). SSD is widespread and rapidly evolving across the animal kingdom such that, depending upon the species, the larger sex can either be male or female [3]. It is not yet clear, however, which mechanisms link the chromosomal sex of an organism to specific male and female patterns of growth during development and thus ultimately to adult SSD.

In mammals, the presence or absence of a single gene on the male Y chromosome (*Sry*) determines gonad differentiation into male testis or female ovary, respectively. The gonads then establish a sex-specific hormonal milieu that controls male and female differentiation, patterns of growth, and metabolism [reviewed in 4,5]. However, recent studies in mice now demonstrate that the cellular (i.e., chromosomal) sex of nongonadal tissues can also influence growth and metabolism, an effect that is thought to be modified by the action of gonadal hormones [reviewed in 6]. Hence, although many details remain to be explored, it is likely in mammals that patterns of growth relevant to SSD are controlled via both hormonal and cell-autonomous mechanisms.

The fruit fly *Drosophila melanogaster* has provided insights into many aspects of sexual dimorphism. In *Drosophila*, sex determination is based on X chromosome dosage, with XX individuals developing as females and XY individuals as males [7,8]. At the early embryonic stage, the presence of 2 X chromosomes in females activates the expression of sex determination gene *Sex-lethal* (*Sxl*), which is thereafter maintained via positive autoregulation [9–11]. *Sxl* controls the splicing of multiple downstream targets regulating sexual differentiation as well as X chromosome dosage compensation [7,8,12]. A commonly held view is that *Drosophila*, unlike mammals, deploys sex determination genes in a strictly cell-autonomous manner such that they are required in every somatic cell that is sexually dimorphic [13,14]. Nevertheless, there are hints that non-cell-autonomous mechanisms regulate at least some aspects of sexual dimorphism. For example, in adult flies, ecdysone and juvenile hormone can act like sex hormones to regulate reproduction and sexual identity [15–17].

SSD in *Drosophila* is present at larval, pupal, and adult stages, with females approximately 30% larger than males [18]. *Drosophila* larvae are composed of 2 types of tissues: polyploid larval-specific organs that are histolysed during pupation and diploid imaginal tissues, which are the precursors of adult structures [19]. Polyploid tissues comprise the bulk of the larva such that body SSD measured at larval stages reflects their growth rather than that of imaginal tissues. In contrast, body SSD measured at the adult stage reflects the larval/pupal growth of imaginal tissues. SSD appears to be present in most tissues and may be a result of differences in both cell size and number [20,21,22, and this study]. Signalling pathways important for body growth, such as the insulin/insulin-like growth factor (IGF) and target of rapamycin (Tor) network [23], are likely to be relevant for SSD but precisely how sex modulates them remains unclear. In terms of sex determination genes, SSD was long thought to be dependent upon *Sxl* but not on one of its key downstream target genes, *transformer* (*tra*), which specifies other aspects of sexual dimorphism [7,11,24]. This was challenged recently by a report that *tra* contributes to SSD, although there is also likely to be a *tra*-independent mechanism [25]. The same study also reported that *tra* not only exerts autonomous effects on cell size but also acts in the female fat body to stimulate the secretion of insulin-like peptides (Ilps) from the brain, which in turn promotes larger body size in females.

Here, we investigate the link between sex determination genes and SSD in *Drosophila* using cell type–specific genetic manipulations and size measurements of both larval and imaginal tissues. We find, surprisingly, that *Sxl* controls SSD by acting in specific subsets of female neurons to increase body growth during larval development. At this stage, neuronal Sxl functions selectively to increase the growth rate of larval but not imaginal tissues. This study reveals that the sex of specific neurons regulates SSD in a non-cell-autonomous manner.

## Results

### SSD is established in L2 without sex-specific systemic Ilp signalling or food intake

**Fig 1:**
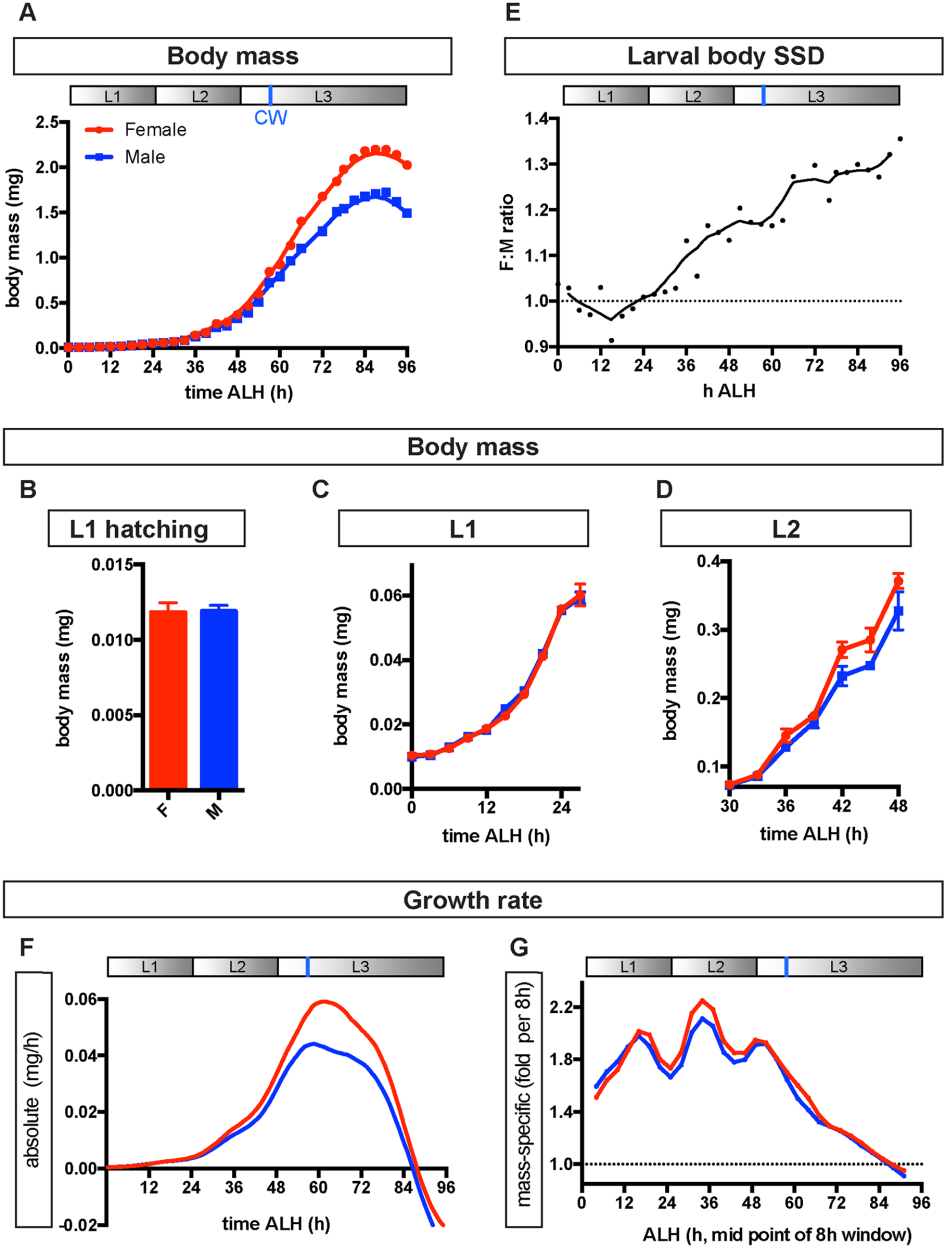
Physiological parameters underlying sexually dimorphic growth. **(A–D)** Growth curve of male and female larvae throughout larval development, showing there is no sex difference in the overall growth period (A), body size at larval hatching (B), or during L1 (C), with a difference in body size first apparent during L2. Graph in (A) shows raw body mass measurements at each time point (dots) and a line of best fit (locally weighted scatterplot smoothing [LOWESS] curve). **(E)** Ratio of female to male (F:M) body mass during larval development calculated from time-course data in (A). Individual points show F:M ratios from raw body mass measurements. Solid line shows F:M ratios calculated from lines of best fit in (A). **(F)** Absolute growth rate throughout larval development, calculated as the slope of the growth curve in (A). **(G)** Mass-specific growth rate throughout larval development, calculated from the data in (A) as a ratio corresponding to the fold growth per unit body mass over 8-h time intervals. Panels A, E, and F show a time line of larval development depicting the 3 instars (L1–L3) and critical weight (CW, vertical blue line). Developmental time, in this and subsequent figures, is measured as hours (h) after larval hatching (ALH). In contrast to this study, previous studies on SSD have focused on the period after CW (see Discussion). The underlying data for this figure can be found in S1 Data.

The *Drosophila* embryo hatches into a larva that develops through 3 instars (L1 to L3), feeding voraciously and increasing its body mass by 2 orders of magnitude. It then enters a long nonfeeding pupal phase, which ends with eclosion of the adult fly. Final body size in *Drosophila* and in other animals is determined by the initial larval size, the growth rate, and the growth period [26]. To understand when SSD first arises during development and which of the 3 parameters contribute to it, we compared the growth curves of male and female larvae. Weighing larvae individually or in small groups at precise times after hatching revealed that the initial sizes of L1 males and females are similar and that SSD only first becomes detectable during L2, thereafter increasing until the female-to-male mass ratio of L3 larvae approaches approximately 1.3 (Fig 1A–1E). Plots of larval mass or of absolute growth rate against time show that the total periods of larval growth in males and females are very similar (Fig 1A and 1F). In contrast, both the absolute growth rate and the mass-specific growth rate (fold growth rate, i.e., growth rate per unit mass) are higher in females than in males (Fig 1F and 1G). Interestingly, sex differences in mass-specific growth rate are greatest during L2, suggesting that mechanisms operating at this stage may make a large contribution towards driving apart the body sizes of males and females (Fig 1G). Together, these results indicate that SSD is established during L2 and involves a higher growth rate in females than in males.

The insulin/IGF family of hormones are known to play a key role in regulating the growth rate in *Drosophila* and other animals [27,28]. Insulin/IGF signalling has also been implicated in SSD in both mammals and *Drosophila* [18,23,25,29]. In *Drosophila*, systemic Ilps are secreted by 7 median neurosecretory cells of the pars intercerebralis, known as insulin-producing cells (IPCs), which are functionally analogous to mammalian pancreatic β cells [30–32]. It has been reported that the retention of insulin-like peptide 2 (Ilp2) in IPCs, which is thought to inversely correlate with insulin secretion [33], is lower in females than in males, and this difference has been suggested to enable increased female growth [25]. However, the Ilp2 measurements in this previous study were made at the late L3 stage, long after we have found that SSD is established. We therefore measured Ilp2 retention in IPCs at the earlier stages of mid-L2 and early L3 but were unable to detect any significant difference between males and females (S1A and S1B Fig). At these early larval stages, we were also unable to detect any significant female increase in 2 key readouts of insulin/IGF-1-like signalling: phosphorylated Akt levels in whole larvae and nuclear exclusion of forkhead box, sub-group O (FoxO) protein in fat body cells (S1C and S1D Fig). Hence, it is unlikely that larval SSD is established by sex-specific differences in the levels of systemic Ilp signalling (at the level of Akt phosphorylation or FoxO nuclear localisation).

We next examined whether the observed sex differences in larval growth rate were associated with food intake. We focused on measuring larval food intake at the early L2 and early L3 stages, when sex differences in growth rate are maximal. We found that the absolute food intake was higher for females than males (S2 Fig). If higher food intake in females drives their increased growth rate, then females would be predicted to have a higher food intake than males per unit of body mass. However, we were unable to detect any significant male-female difference in food intake per unit of body mass (mass-specific food intake) at either early L2 or early L3 (S2 Fig). We cannot rule out that very small differences in mass-specific food intake do exist between males and females but are hard to detect. Nevertheless, these results suggest that sex differences in absolute food intake are a consequence of preexisting body size differences rather than the driving force that establishes SSD.

### *Sxl* acts in the nervous system to control SSD of the larval body

**Fig 2:**
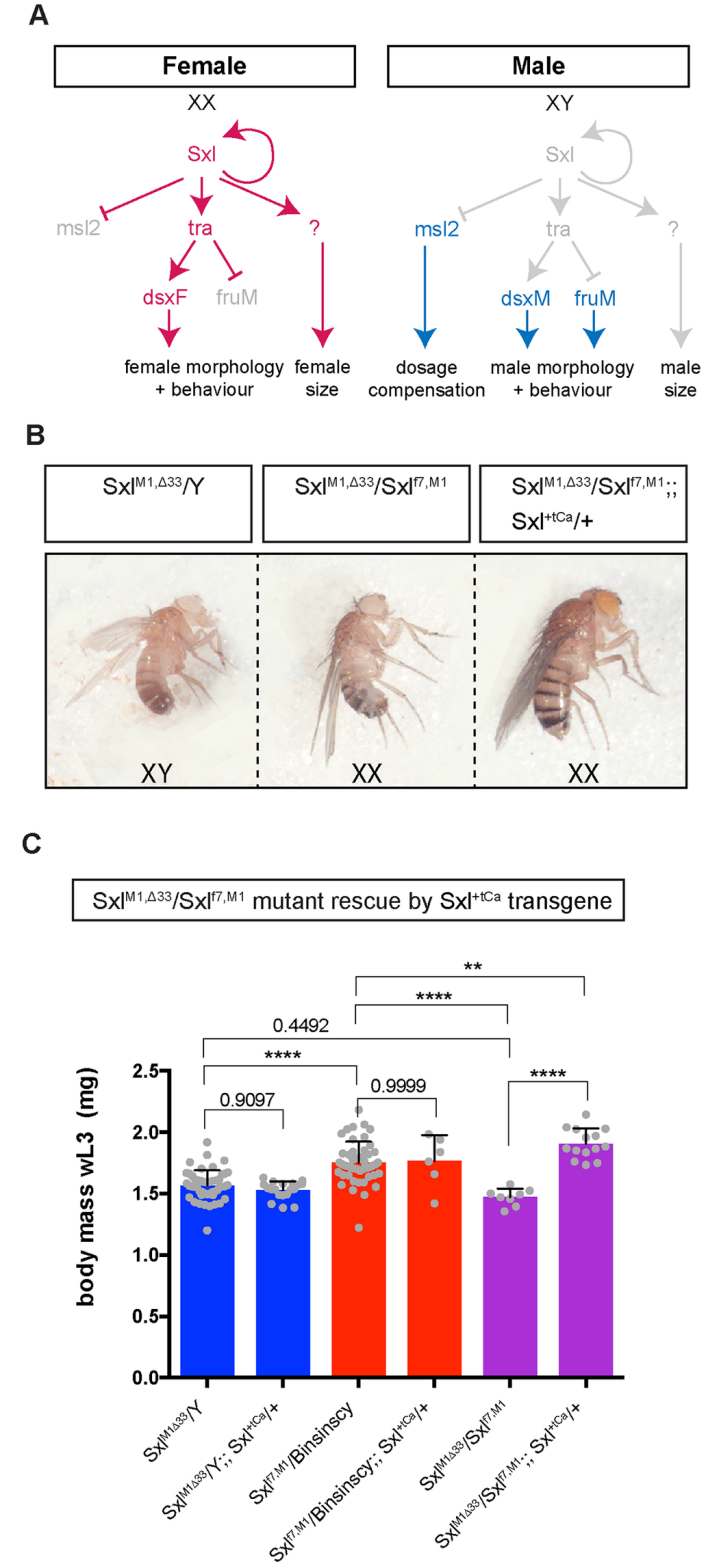
*Sxl* controls the sexual size dimorphism (SSD) of the larval body. **(A)** Schematic of the sex determination pathway in *Drosophila* and its control of sexual differentiation and dosage compensation. Genes/proteins active in females are displayed in red; genes/proteins active in males are displayed in blue. **(B)** Transheterozygous *Sxl*^*M1*, *Δ33*^*/Sxl*^*f7*, *M1*^ mutant female adults are masculinised in terms of morphology and body size (middle panel). Both morphology and body size can be rescued by a wild-type copy of the *Sex-lethal* (*Sxl*) gene, *Sxl*^*+tCa*^ (right panel). **(C)** *Sxl*^*M1*, *Δ33*^*/Sxl*^*f7*, *M1*^ mutant female larvae are also masculinised in terms of larval body mass, and this can be rescued by *Sxl*^*+tCa*^, showing that *Sxl* functions to control larval SSD. Individual data points, means, and SD are plotted. Asterisks denote significant changes according to a 1-way ANOVA with multiple comparisons (** *p* < 0.01, **** *p* < 0.0001), otherwise *P* values >0.05 are shown. The underlying data for this figure can be found in S1 Data.

To address how chromosomal sex regulates larval SSD, we first tested the function of *Sxl*, an upstream component of the sex determination pathway required in females (Fig 2A). We made use of 2 specific mutant alleles of *Sxl* that show partial female viability as they retain some ability to repress *male-specific lethal 2* (*msl-2*) expression and therefore avoid female-lethal levels of ectopic dosage compensation [11,24]. Transheterozygous *Sxl*^*M1*, *Δ33*^*/Sxl*^*f7*, *M1*^ females are masculinised not only in terms of their adult size and morphology but also their larval body size (Fig 2B and 2C). Furthermore, all 3 parameters can be rescued back to the female state by the addition of 1 wild-type copy of *Sxl* (Fig 2B and 2C).

**Fig 3:**
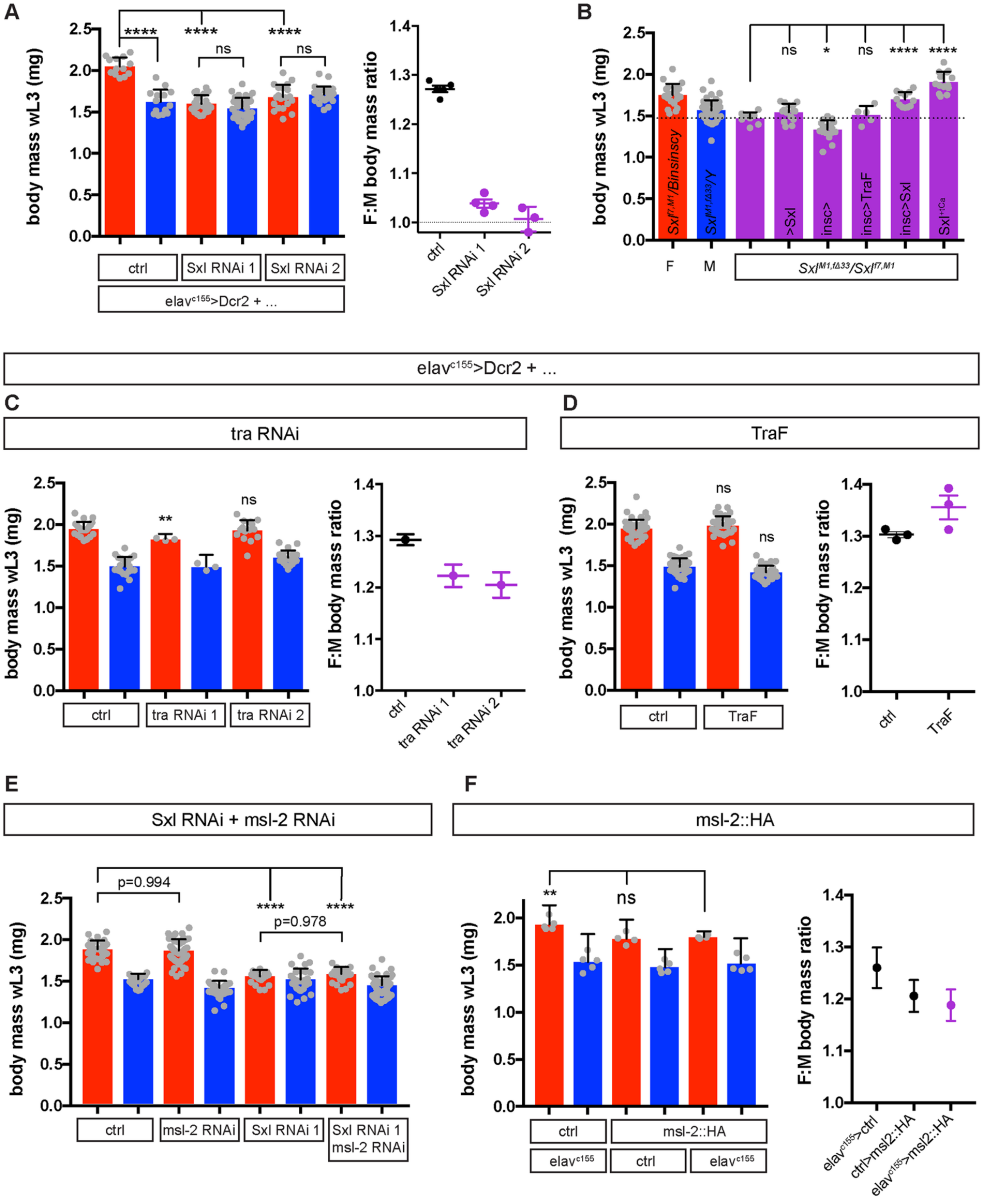
Sxl functions in the nervous system to control larval sexual size dimorphism (SSD). **(A)** RNAi-mediated knockdown of *Sex-lethal* (*Sxl*) in all neurons (*elav*^*c155*^*-Gal4*) specifically reduces female body mass in wandering L3 larvae (wL3). This abolishes sex differences in body mass, leading to a female to male ratio (F:M ratio) near 1. Body mass data shows representative results of 3 independent experiments, F:M ratio data shows mean ratio, individual ratios, and SEM of 3–4 independent experiments. **(B)** Rescue of wL3 body mass of Sxl^M1, Δ33^/Sxl^f7, M1^ mutant females by neuronal expression of upstream activation sequence (UAS)-Sxl (*Insc>Sxl*) but not of the female splice variant of *transformer*, UAS-TraF (*Insc>TraF*). **(C–D)** Pan-neuronal knockdown of *transformer (tra)* (C) or overexpression of the female-specific *tra* splice variant (TraF) (D) has minor or no effects on body mass and SSD. In (C), tra RNAi 1 individual points represent means from groups of 7–18 larvae each. For (D), body mass data show representative results of 3 independent experiments, F:M ratio data plots mean ratio, individual ratios, and SEM of 3–4 independent experiments. **(E)** *Male-specific lethal 2* (*msl-2*) knockdown does not rescue the female body mass of *elav*^*c155*^*>Sxl RNAi* larvae (genotype is *elav*^*c155*^*>Sxl RNAi 1 + msl-2 RNAi*). Note that *elav*^*c155*^*>msl-2 RNAi* has no effect on female body mass (*p* = 0.994) but leads to a very small decrease in male body mass with or without *Sxl RNAi* (*p* = 0.0028 and *p* = 0.049, respectively). Graph shows results representative of 3 independent experiments. **(F)** Neuronal expression of Msl-2 (*elav*^*c155*^*>Msl-2*::*HA*) does not significantly decrease female body mass. Individual points represent means from groups of 10 larvae. Unless otherwise noted, graphs of body mass plot mean, SD, and individual data points, and F:M ratio graphs plot the mean ratio of female to male body mass and SEM. * *p* < 0.05, ** *p* < 0.01, and **** *p* < 0.0001 according to 1-way ANOVA with multiple comparisons. The underlying data for this figure can be found in S1 Data.

To understand in which tissues Sxl acts to control SSD, we switched the sex of individual organs from female to male by expressing *upstream activation sequence (UAS)-Sxl RNAi* with tissue-specific Gal4 drivers. Previous work implicated the sex of the fat body in SSD, mediated via the *transformer (tra)* branch of the sex determination pathway [25]. Surprisingly, we observed that fat body–specific RNAi knockdowns of *tra* or its partner *transformer 2* (*tra2*) or even *Sxl* only led at best to minor reductions in female body size and SSD, quantified by the female/male body mass ratio (S3 Fig). We next knocked down *Sxl* in 2 larval cell types whose adult counterparts are known to have sexually dimorphic functions, the midgut enterocytes and the oenocytes [16,34–36], but this gave no significant alterations in larval SSD (S4 Fig). Strikingly, however, using a pan-neuronal Gal4 driver (*elav*^*c155*^*-Gal4*) to knock down *Sxl* with either of 2 UAS-RNAi lines (*UAS-Sxl RNAi 1* or *UAS-Sxl RNAi 2*, see Materials and methods) led to a strong and specific decrease in the body size of females, completely abrogating SSD (Fig 3A). In the reciprocal experiment, nervous system–specific expression of a *UAS-Sxl* mRNA [24] (driven by *Insc-Gal4*) was sufficient to rescue significantly the larval body size of *Sxl* mutant females, although not as efficiently as with *Sxl*^*+tCa*^, a complete copy of the *Sxl* gene containing its own regulatory elements (Fig 3B). Together, these experiments indicate that neural *Sxl* activity is both necessary and, at least in part, sufficient for larval SSD. Tracking the growth of *elav*^*c155*^*>Sxl RNAi* (elavc155-Gal4; UAS-Sxl RNAi) female larvae throughout L2 revealed that they grow at the same rate as males (S5 Fig). Thus, neuronal Sxl is required for the establishment of larval body SSD as well as for its subsequent maintenance. Interestingly, *elav*^*c155*^*-Gal4* driven RNAi for *tra* or overexpression of *TraF*, the female-specific *tra* splice variant, had little or no significant effect upon larval SSD (Fig 3C and 3D). Furthermore, although the body mass of *Sxl* mutant females was rescued significantly by *Insc-Gal4*–mediated expression of Sxl, this was not the case for TraF (Fig 3B). This provides evidence that neural Sxl regulates larval SSD largely independently of its characterised downstream target, *tra*.

**Fig 4:**
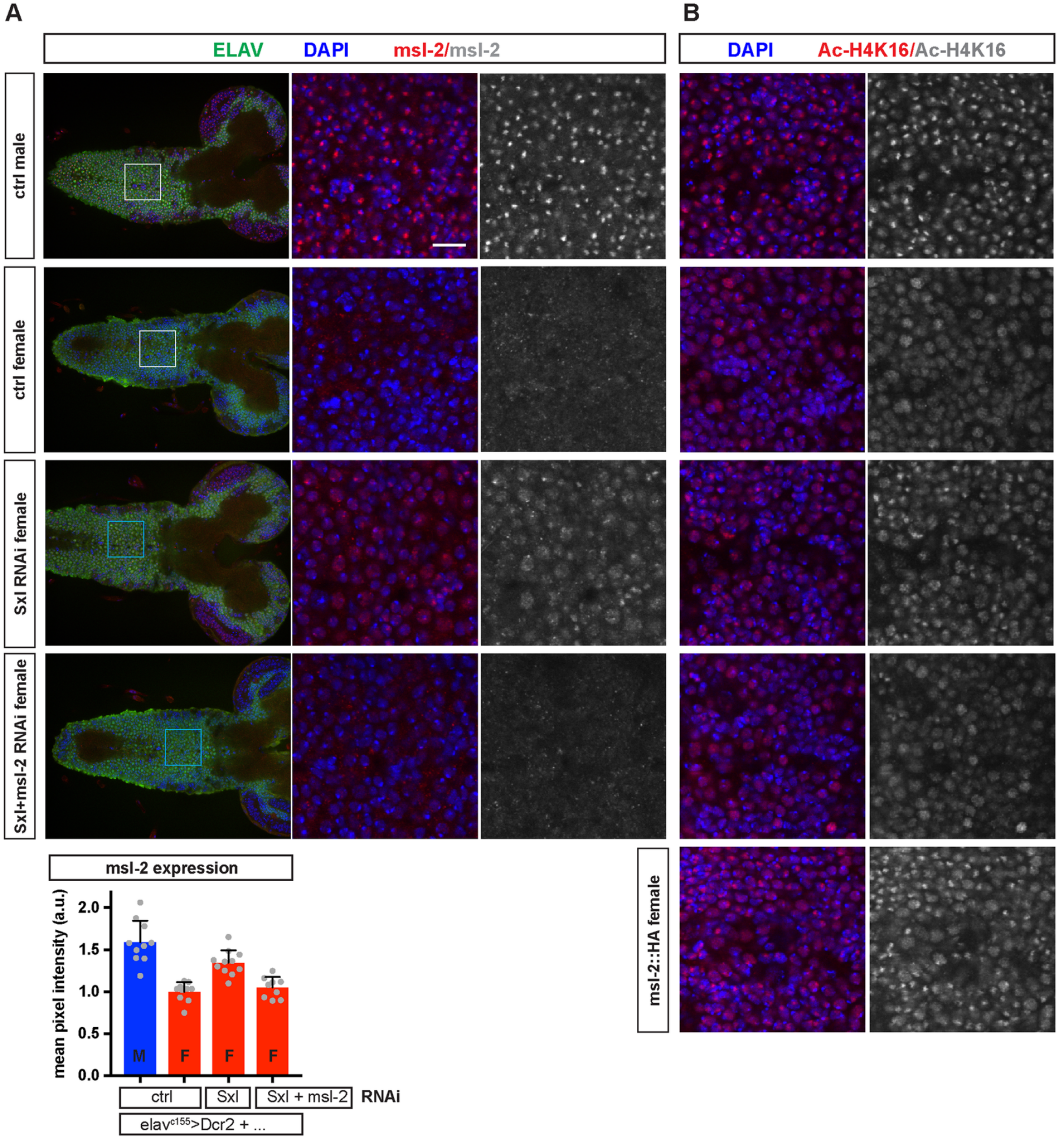
Male-specific lethal 2 (*msl-2*) knockdown blocks expression of Msl-2 and acetylated histone H4 lys16 (H4K16) in *elav*^*c155*^*>Sxl RNAi* females. **(A)** Expression of Msl-2 and Elav (a neuronal marker) with DNA stained by DAPI in central nervous systems (CNSs) from early L3 larvae of the indicated sex and genotype. Left panels show single confocal sections close to the neuropil, where functional neurons, not immature postembryonic neurons, predominate. Centre and right panels show higher magnification views of boxed regions in the left panels. Msl-2 in control males localises to nuclear foci, consistent with its X chromosome localisation, and it is weak/absent in control females. In *elav*^*c155*^*>Dcr2 + Sxl RNAi 1 + msl-2 RNAi* females, Msl-2 is expressed at moderate levels, and this ectopic Msl-2 is suppressed in *elav*^*c155*^*>Dcr2 + Sxl RNAi 1* females. Bottom graph shows quantification of Msl-2 expression (mean fluorescence intensity) measured in a region of interest similar to the boxed regions. Note that mean fluorescence intensity levels in control female levels likely reflect background signal. **(B)** Expression of histone H4 acetylated at lysine 16 (Ac-H4K16) with DNA stained by DAPI in CNSs from early L3 larvae of the indicated sex and genotype. Left and right panels show high magnification views from similar regions of the CNS as shown in A. Ac-H4K16 in control males strongly localises to nuclear foci (the X chromosome) and, in both sexes, lower signal intensity is observed throughout the nucleus (autosomes). Ac-H4K16 nuclear foci are observed in *elav*^*c155*^*>Dcr2 + msl-2*::*HA* and *elav*^*c155*^*>Dcr2 + Sxl RNAi 1* females but not in *elav*^*c155*^*>Dcr2 + Sxl RNAi 1 + msl-2 RNAi* females. The underlying data for this figure can be found in S1 Data.

An important function of Sxl is to repress at the translational level the expression of *msl-2*. *msl-2* is required in males for X chromosome dosage compensation (Fig 2A). This raises the possibility that inappropriate dosage compensation due to neural up-regulation of Msl-2 in *elav*^*c155*^*>Sxl RNAi* females might produce “sickness” effects that inhibit larval growth. However, 3 lines of evidence rule out this possibility. First, we showed that the growth trajectory of *elav*^*c155*^*>Sxl RNAi* females precisely matches that of control males, making it unlikely that this is due to a nonspecific sickness effect on growth (S5 Fig). Second, we find that neural *msl-2* knockdown in *elav*^*c155*^*>Sxl RNAi + msl-2 RNAi* females does not decrease the strength of the *Sxl* knockdown (S6 Fig). In this context, *msl-2* knockdown efficiently blocks up-regulation of Msl-2 and a marker of X chromosome dosage compensation (histone H4 lys16 [H4K16] acetylation) but, importantly, it does not rescue body mass (Figs 3E, 4A and 4B). And third, neural misexpression of Msl-2 (*elav*^*c155*^*>msl-2*::*HA*) in females does not decrease body mass, yet it does induce neuronal H4K16 acetylation to a level at least as high as that observed in *elav*^*c155*^*>Sxl RNAi* females (Figs 3F and 4B). Hence, inappropriate neural expression of the Msl-2 dosage compensation pathway does not account for the masculinization of body growth observed in *elav*^*c155*^*>Sxl RNAi* females. Together, the results thus far demonstrate that Sxl acts in the female nervous system to regulate larval body SSD in a non-cell-autonomous manner.

### Larval SSD requires Sxl in IPCs and in Gad1-Gal4 neurons

**Fig 5:**
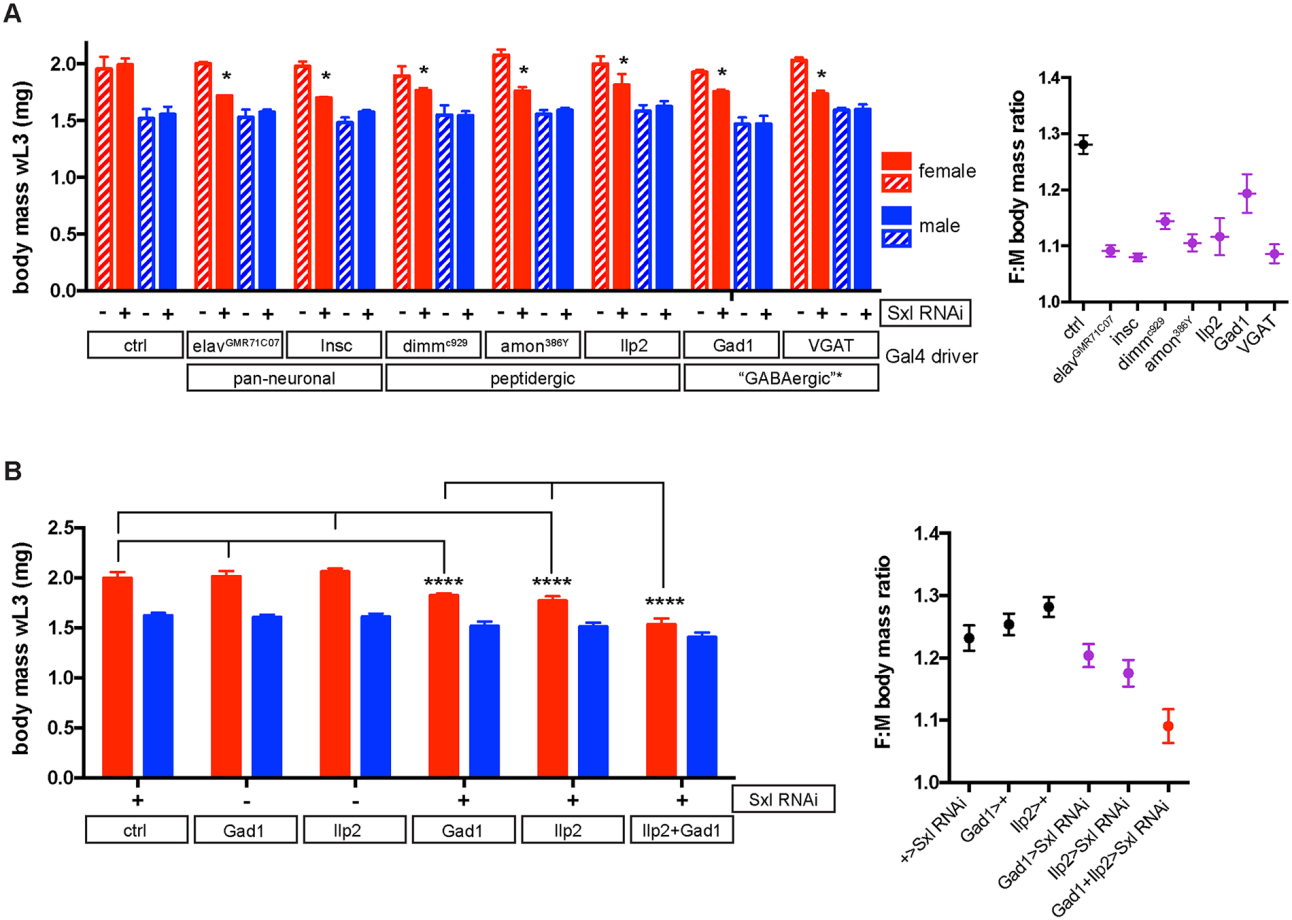
*Sex-lethal* (*Sxl*) is required in insulin-producing cells (IPCs) and Gad1-Gal4 neurons to control sexual size dimorphism (SSD). **(A)** Gal4-driver screen identifies 7 pan-neuronal, broad peptidergic (*dimm*^*c929*^, *amon*^*386Y*^), IPC (insulin-like peptide 2 [*Ilp2*]), and GABAergic* drivers with female-specific effects on body mass when combined with *upstream activation sequence* (*UAS*)*-Sxl RNAi*. For details and full results of screen, see S8 Fig. Left graph shows mean body mass and SD of 2–16 replicates of groups of 3–11 larvae. Hatched bars depict controls lacking the Sxl RNAi transgene, identified by the *CyO*, *Dfd-YFP* balancer, except for *Gad1-Gal4* controls, which also include *CyO*, *Dfd-YFP>Sxl RNAi* larvae. In all cases, * *p* < 0.001 from respective no-driver controls using 2-way ANOVA with multiple comparisons. Right graph shows mean female to male (F:M) body mass ratios and SEMs for the Gal4 driver hits that decrease larval SSD. Expression analysis of *elav*^*c155*^*-Gal4* and the other driver hits in this screen suggests that there is no shared secondary site of larval expression outside the nervous system (S1 Table). *Note that the 2 “GABAergic” drivers (*Gad1-Gal4* and *VGAT-Gal4*) show only partially overlapping expression with GABA+ neurons (see S1 and S2 Images). **(B)** *Ilp2-Gal4* and *Gad1-Gal4* act additively to decrease female larval body mass via *Sxl* knockdown. Left graph shows mean body mass and SD of 3–4 replicates of groups of 6–10 larvae. Right graph shows mean F:M body mass ratios and SEMs. Knockdown of *Sxl* using both *Ilp2-GAL4* and *Gad1-Gal4* decreases female body mass and SSD more strongly than with each driver alone. Note that for *Ilp2>Sxl RNAi* and *Ilp2 + Gad1>Sxl RNAi* genotypes, results were pooled from 3 independent recombinants of *Ilp2-Gal4*, *UAS-Sxl RNAi 1*, each crossed to a no-driver control or to *Gad1-Gal4*, respectively. **** indicates *p* < 0.0001 using 1-way ANOVA with multiple comparisons. The underlying data for this figure can be found in S1 Data.

We next determined which cell types in the nervous system require the Sxl activity relevant for larval SSD. Elav is a well-established pan-neuronal marker but it is also reported to be transiently expressed in glia [37]. However, knockdown in glia does not account for the abolition of SSD in *elav*^*c155*^*>Sxl RNAi* larvae (S7 Fig). To identify the neuronal population(s) in which Sxl is required to control larval SSD, we screened a panel of 33 Gal4 drivers expressed in different neuronal subsets (S8 Fig and Fig 5A). Two additional pan-neuronal drivers (*Insc-Gal4* and *elav*^*GMR71C07*^*-Gal4*) as well as five neuronal subset drivers crossed to *UAS-Sxl RNAi* gave a significant decrease in female but not male larval body size (S8 Fig and Fig 5A). The 5 neuronal subset drivers fall into 2 categories: those expressed in peptidergic neurons (*dimm*^*c929*^*-Gal4*, *amon*^*386Y*^*-Gal4*, and *Ilp2-Gal4*) and those expressed in GABAergic neurons (*Gad1-Gal4* and *VGAT-Gal4*). However, we find that both “GABAergic” drivers are expressed in different patterns in the CNS of both early and late L3 larvae and they only partially overlap with GABA-positive neurons (S9 Fig, S1 and S2 Images).

Given that we did not find sex differences in insulin signalling during larval SSD establishment, it is intriguing that our Gal4 screen for SSD neurons identified *Ilp2-Gal4*, which is expressed in IPCs. However, *Ilp2-Gal4* is also weakly expressed in some neurons of the ventral nerve cord (VNC) [38]. To pinpoint whether IPC or VNC neurons are relevant for SSD, the Gal4 activity of *Ilp2-Gal4* was inhibited specifically in IPCs by driving the expression of *LexAop-Gal80* using *Ilp2-lexA*, which is not expressed in VNC neurons (S10 Fig). This combined Gal4/LexA manipulation restored Sxl expression specifically in IPCs and also rescued the SSD of *Ilp2>Sxl RNAi* larvae (S11 Fig). We also carried out a reciprocal experiment, restoring Sxl in VNC neurons but not in IPCs using *tsh-Gal80*, which efficiently suppresses Gal4 driver activity in the VNC (S12A Fig). However, this did not rescue the SSD of larvae expressing *Sxl RNAi* under the control of *Ilp215-1-Gal4*, a driver expressed in both IPCs and VNC neurons (S10 and S12B Figs). Together, these 2 experiments provide important controls demonstrating that Sxl acts in IPCs to promote SSD.

We next investigated the surprising finding that, despite a clear role for the IPCs, insulin signalling itself does not seem to be involved in larval body SSD. To override any potential sex differences in the transcription of endogenous Ilps that might not have been detected, Ilp2 or Ilp5 were specifically overexpressed within IPCs but neither manipulation affected SSD (S13A Fig). In addition, PI3-kinase activity was blocked specifically in IPCs (*Ilp2+Ilp5>p60*), which decreases larval body size probably via down-regulation of Ilp production [39,40], but this also failed to decrease SSD significantly (S13B Fig). Furthermore, there is little or no change in larval SSD following loss of Ilp2 activity (*Ilp2*^*1*^ mutants) or deletion of all Ilps normally expressed in IPCs (*Df(Ilp1-5)* mutants), even though the body size of both sexes is strongly decreased (S13D Fig). Surprisingly, therefore, the role of IPCs in larval body SSD is dependent upon the activity of *Sxl* but apparently not Ilps.

Unlike *Ilp2-Gal4*, the other Gal4 drivers that gave a hit in our SSD screen are each expressed in large subpopulations of neurons. The effects of *dimm*^*c929*^*-Gal4*, *amon*^*386Y*^*-Gal4*, and *VGAT-Gal4* are all likely to involve their expression overlapping in IPCs ([41] and S14A and S14B Fig). Consistent with but not proving this, blockade of Gal4 activity throughout a large neural domain that does not contain the IPCs, the VNC (using *tsh-Gal80*), does not suppress the ability of *VGAT-Gal4* or *amon*^*386Y*^*-Gal4* to decrease SSD when driving Sxl RNAi (S12B Fig). Another driver hit in our SSD screen is *Gad1-Gal4* and this is not expressed in IPCs (S14B Fig). This finding and the observation that neither *Gad1-Gal4* nor IPC drivers decrease female-specific body size as completely as pan-neuronal drivers suggested that Sxl could be required additively in 2 distinct subpopulations of neurons. To test this, *Ilp2-Gal4* and *Gad1-Gal4* drivers were combined to knock down Sxl in both subsets of neurons, and this gave a stronger decrease in female body size than was observed with either driver alone (Fig 5B). The combination of *Ilp2-Gal4* and *Gad1-Gal4* decreases SSD strongly to a value approaching that obtained with pan-neuronal drivers (*elav*^*c155*^*-Gal4* or *Insc-Gal4*). We therefore conclude that Sxl acts additively in at least 2 distinct neural subsets, IPCs and *Gad1-Gal4* neurons, to control larval SSD via a novel mechanism that does not require Ilp regulation.

### Different mechanisms regulate SSD in larval and imaginal tissues

**Fig 6:**
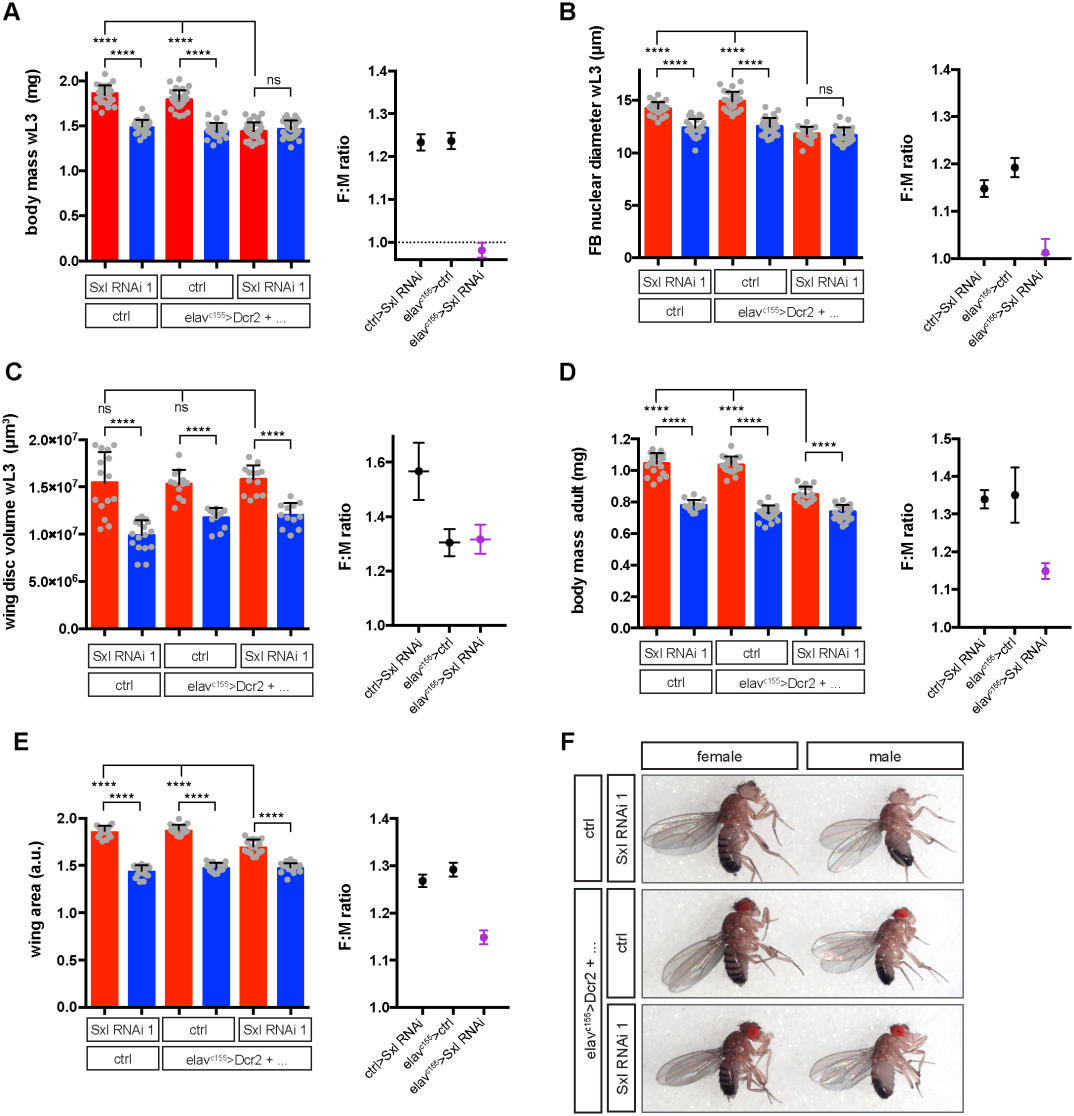
Neuronal Sex-lethal (Sxl) selectively controls the sexual size dimorphism (SSD) of larval versus imaginal tissues. Effect of neuron-specific depletion of Sxl (*elav*^*c155*^*>Sxl RNAi 1*) on SSD of the body and different tissues at larval and adult stages. For panels A–E, the histograms on the left show the male and female body or tissue sizes used to calculate the female to male (F:M) ratios in the graph on the right. **(A)** SSD of body mass in wandering L3 larvae (wL3) is abolished in *elav*^*c155*^*>Sxl RNAi* animals. **(B)** SSD of fat body nuclei diameter (a proxy for cell size) in wL3 larvae is abolished in *elav*^*c155*^*>Sxl RNAi* animals. **(C)** SSD of wing disc volume in wL3 larvae is not decreased in *elav*^*c155*^*>Sxl RNAi* animals. Note that 1 of 2 control genotypes has an abnormally high F:M ratio of approximately 1.6, but this is due to a decrease in male size not to a change in female size. **(D)** SSD of adult body mass is decreased but not abolished in *elav*^*c155*^*>Sxl RNAi* animals. **(E)** SSD of adult wing area is decreased but not abolished in *elav*^*c155*^*>Sxl RNAi* animals. This contrasts with lack of an SSD effect in wing discs at the end of larval development **(C)**. **(F)** Images of adult flies, showing that *elav*^*c155*^*>Sxl RNAi* decreases female body size without altering female-specific pigmentation of the abdominal cuticle. For adult body and tissue measurements **(D–E)**, animals were transferred to 18°C from pupariation to adulthood to improve adult viability and analysed 1–2 d posteclosion. All graphs of body or tissue measurements show mean, SD, and individual data points. * *p* < 0.05, ** *p* < 0.01, and **** *p* < 0.0001 using 1-way ANOVA with multiple comparisons. All graphs of F:M ratios show mean and SEM. The underlying data for this figure can be found in S1 Data.

The finding that neuronal Sxl regulates female larval body size appears to be at odds with the widely held view that somatic sex determination in *Drosophila* is a cell-autonomous process. Early evidence for the cell autonomy of sexual dimorphism, including SSD, came from studies of gynandromorphs, mostly XX/XO mosaic adults [42]. However, the external adult structures that were the focus of the gynandromorph study are derived from diploid imaginal cells, not the polyploid cells that constitute the bulk of the larval body mass measured in our experiments. We therefore compared the cell-autonomous requirements of sex determination genes for SSD of the wing disc versus the fat body, i.e., a diploid imaginal versus a polyploid larval tissue, respectively. In a control genetic background, both the wing disc and the fat body are clearly larger in females than males by the end of larval development (see Fig 6B and 6C). Manipulating sex determination genes in the pouch region of the imaginal wing disc (using *nubbin-Gal4*), we observed sex-biased changes in the size of the pouch relative to the whole wing disc (S15A Fig). Measuring the female to male ratio of this parameter revealed modest reductions in wing pouch SSD with *Sxl RNAi*, *tra RNAi*, or TraF overexpression (S15B Fig). Similarly, expressing *Sxl RNAi* or *tra RNAi* in larval fat body cells (using Flp-out clones, see Materials and methods), gave small reductions in the nuclear diameter of female but not male fat body cells (S15C Fig). These findings are broadly similar to those in a published study [25] and together indicate that sex determination genes make a cell-autonomous contribution to the SSD of both larval and imaginal tissues. However, for the larval fat body, we note that Sxl/tra cannot significantly increase cell size in an autonomous manner in 2 different contexts lacking neuronal Sxl function: males misexpressing TraF in fat body clones and *elav*^*c155*^*>Sxl RNAi* females (S15C Fig and Fig 6B).

We next compared the non-cell-autonomous requirement of neuronal Sxl for SSD in imaginal versus larval tissues. Remarkably, the larger size of the female wing imaginal disc remains unaffected in *elav*^*c155*^*>Sxl RNAi* larvae, despite the decreased body mass and fat body nuclear size typical of males (Fig 6A–6C). Thus, neuronal *Sxl* activity is not required for the SSD of a developing imaginal tissue, in contrast to that of a developing larval tissue. However, examination of adult *elav*^*c155>*^*Sxl RNAi* flies revealed a significant strong decrease but not a complete loss of SSD for body mass and for wing area (Fig 6D–6F). Together with our previous results, this shows that neuronal *Sxl* activity is not required for the SSD of developing imaginal tissues during larval stages but, during subsequent pupal stages, it does contribute to the final SSD of adult body structures. Interestingly, we also noticed differences in the role of insulin signalling for the SSD of larval versus imaginal tissues. Hence, a genetic manipulation that decreases Ilp production from IPCs (*Ilp2+Ilp215-3>p60*) reduces absolute body size in both sexes without altering larval body SSD (S13B Fig). In contrast, the same manipulation reduces the size of the wing imaginal disc from female but not male larvae and, strikingly, this completely abolishes wing disc SSD (S13C Fig). Furthermore, knockdown of the *Drosophila* insulin receptor (InR) in the wing disc pouch (*nubbin>InR RNAi*) not only reduced wing pouch size relative to the whole wing disc in both male and female larvae but it also decreased wing pouch SSD (S15A and S15B Fig). These results together raise the possibility that Sxl may boost female-specific imaginal but not larval tissue growth, at least in part by increasing its sensitivity to systemic Ilps.

Overall, we conclude that, during larval development, different mechanisms control the sex-specific growth of larval versus imaginal tissues. In the final adult, SSD is a collective function of neuronal *Sxl* that has acted in a non-cell-autonomous manner on larval tissues as well as *Sxl* and *tra* functioning in a cell-autonomous fashion in both larval and imaginal tissues.

## Discussion

This study uncovers the existence of a new physiological mode of action for sex determination genes in the specification of *Drosophila* body size. It demonstrates that the female sex determination gene *Sxl* acts in specific groups of neurons as a remote stimulator of body growth. It also shows that the sex determination machinery utilizes different mechanisms for regulating the size of the larval versus the adult body morphs. A key conclusion here is that Sxl acts in neurons to control primarily the growth of the larval morph. We now discuss the implications of these findings, how they support a new neuronal relay model for SSD, and how they reveal that mechanisms of sexual differentiation in *Drosophila* and mammals may be more similar than previously thought.

### Larval SSD is established early and without sex-specific insulin signalling

We found that sex-specific body sizes are established after embryogenesis but early during larval development. SSD is manifested as a higher growth rate in female than male larvae, with a maximal difference occurring during L2 and early L3. Previous SSD studies focused later in development, after critical weight (CW), a key checkpoint in L3 that can influence final body size [18,23,25]. The study by Testa et al. [18] showed that females are larger as they attain a higher CW and then a higher absolute growth rate during subsequent larval development (the terminal growth period). Our data are consistent with this but also reveal that the establishment of different growth patterns in females and males involves an early divergence of mass-specific growth rates (mass gain per h per mg of body mass) in L2, well before CW. After SSD has been established in L2/early L3, it is subsequently maintained as larger female larvae gain more absolute mass per hour than smaller males, although both sexes now have very similar mass-specific growth rates.

The insulin signalling pathway is a major stimulator of larval growth in *Drosophila* and it has been reported that IPC secretion of Ilp2 and InR signalling are both higher in females than in males at the L3 stage [25]. However, at earlier stages relevant for the establishment of SSD, we were unable to detect any male-female differences in Ilp2 secretion or in insulin signalling. Furthermore, larvae with decreased Ilp production from IPCs or larvae lacking all 3 of the Ilp genes normally expressed in the IPCs are smaller but still retain larval body SSD. Thus, insulin signalling is required for maximal larval growth in both males and females but it does not appear to be a sex-specific regulator of larval SSD. Interestingly, however, SSD of the developing wing disc is decreased by InR knockdown or decreased insulin production from IPCs. Thus, although insulin signalling may not contribute to the SSD of larval tissues, it does regulate that of imaginal tissues. Higher insulin signalling in female imaginal tissues could be driven by local Sxl/tra dependent modulation of insulin sensitivity and perhaps from mid/late L3 stages by increased Ilp production [25]. Very recently, it was reported that another key growth regulator, Myc, is more highly expressed in female than in male larvae and so may contribute to SSD [43]. Sex differences in *myc* transcript expression may be the result of this X-linked gene escaping complete dosage compensation rather than via direct Sxl regulation. Interestingly, we find that the higher expression of *myc* characteristic of females is fully maintained in *elav*^*c155*^*>Sxl RNAi* larvae, even though they are masculinised in terms of larval growth (S15 Fig). This indicates that a sex difference in global *myc* levels is not sufficient to confer SSD. It remains possible, however, that *myc* in neurons or in another tissue has a role in SSD, potentially downstream of neuronal Sxl.

### Sxl acts in IPCs and Gad1-Gal4 neurons to control larval SSD

A central finding of this study is that Sxl is required in the CNS to direct the female-specific growth trajectory of the larval body. By mapping the site of action of Sxl in the CNS using a panel of Gal4 drivers, we were able show that it functions additively in 2 nonoverlapping populations of neurons within the CNS: *Gad1-Gal4* neurons and IPCs. For the *Gad1-Gal4* neurons, it remains to be determined whether GABA^+^ or GABA^-^ subsets are relevant for SSD and whether or not they functionally interconnect with IPCs in the larval CNS. Nevertheless, it has been reported for the adult CNS that some GABAergic neurons converge on IPCs, which express the metabotropic GABA_B_ receptor and may respond to GABA by decreasing Ilp secretion [44,45]. Importantly, our study also mapped another critical site of action for Sxl to the IPCs, a cluster of only 7 peptidergic neurons. This finding, together with the evidence that sex-specific IPC secretion of Ilps is unlikely to establish SSD, suggests that 1 or more of the numerous other neuropeptides/secreted factors expressed in larval IPCs may be relevant [46].

We found that neuronal Sxl appears to regulate SSD largely independently of its best-characterised downstream targets, *tra* and *msl-2*. The lack of a major neuronal *tra* input in SSD makes it unlikely that there is a role for its neuronal target *fruitless*, which regulates many aspects of sex-specific behaviour in adults [47,48]. In addition, sex-specific isoforms of Fruitless have not been detected until the late L3 stage [49], long after SSD has been established. New direct Sxl targets have been identified in recent years in several biological contexts [50–54] and future approaches targeting the early larval nervous system may reveal how neuronal Sxl controls SSD. Importantly, whatever the Sxl targets in neurons relevant for SSD turn out to be, our results clearly demonstrate that Sxl acts in a remote manner to regulate peripheral tissue growth and overall body size. Identification of the complete remote-control pathway is beyond the scope of our study but, in principle, it could involve the neural secretion of a hormone-like signal and/or innervation of peripheral organ(s) that regulate growth.

### A neuronal relay model for the control of sex-specific growth in *Drosophila*

A previous report implicated the Sxl target *tra* acting in the fat body as an important non-cell-autonomous regulator of female body size [25]. Our study, however, finds that the genetic requirement for *Sxl* in body size is stronger in neurons (likely acting without *tra*) than it is in the fat body (acting with *tra*). Not all of the conclusions made in the 2 studies are easily reconcilable, but we note that the previous report used body size readouts that were adult mass or estimated pupal volume, whereas we measured larval body mass during L2 and L3. It nevertheless remains possible that Sxl can act in both neurons and fat body to regulate overall body SSD in a non-tissue-autonomous manner. Perhaps the relative contributions of each site of Sxl expression depend upon diet or other factors that varied between the 2 studies. Either way, our finding that restoration of Sxl expression specifically in neurons is sufficient to increase the body size of *Sxl* mutant females demonstrates that the neuronal relay mechanism is functional without Sxl in the fat body.

**Fig 7:**
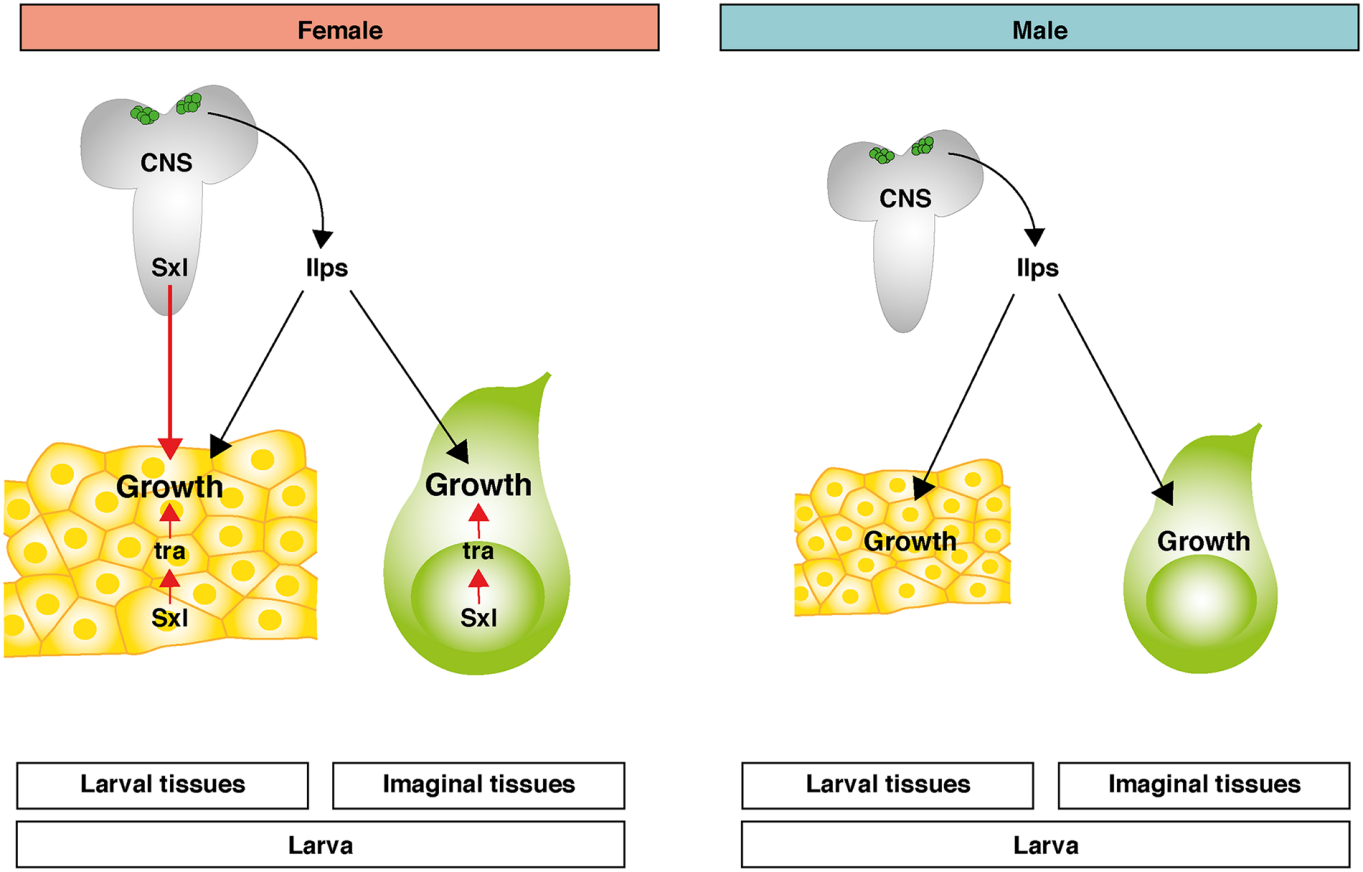
A relay model for the neuronal control of sexual size dimorphism (SSD) in *Drosophila*. Sex-lethal (Sxl) expression in insulin-producing cells (IPCs) and other neurons of the central nervous system (CNS) acts, largely independently of the female-specific *tranformer* splice variant (TraF), to relay a signal(s) to peripheral larval tissues. This signal specifies the female-specific growth trajectory of larval tissues and does not appear to involve IPC-derived insulin-like peptides (Ilps) or insulin signalling. Imaginal tissue growth during larval stages is insensitive to the neuronal Sxl signal. Sxl also acts cell-autonomously via TraF in both larval and imaginal tissues to increase female growth. Red arrows indicate female-specific regulation; black arrows indicate non-sex-specific growth regulatory pathways. Additional SSD mechanisms not depicted in this model are proposed in other studies [25,43].

We now discuss the evidence supporting a neuronal relay model for SSD in *Drosophila* (Fig 7). Central to this model is the finding that Sxl in neurons is required to relay a signal for female body size to peripheral larval tissues. Thus, loss of Sxl in neurons abolishes SSD by decreasing the larval body mass of females to that of males. Conversely, neuronal Sxl expression is sufficient for substantial rescue of female body size in *Sxl* mutant larvae. Neuronal *Sxl* knockdown decreases SSD in larvae at the level of individual polyploid larval tissues such as the fat body, but, surprisingly, this does not appear to be the case for the diploid wing imaginal disc. Nevertheless, after completion of pupal development, neuronal *Sxl* depletion does eventually lead to decreased SSD of the adult wings as well as the adult body. So, how can we account for why the neuronal Sxl input affects imaginal tissue SSD at adult but not larval stages? One possible explanation is that neuronal Sxl functions during pupal stages to nonautonomously regulate imaginal tissue growth. Alternative explanations involve neuronal Sxl acting during larval stages to specify pupal resources/signals that themselves regulate the SSD of adult body structures. For example, nutrient resources laid down in larval tissues can be mobilized by the process of histolysis during the pupal period [55]. Hence, the greater mass of the female larva may be needed to sustain the greater final size of the female adult. An important feature of our relay model for SSD is that the Sxl-dependant signal from neurons is integrated with local *Sxl/tra* inputs in both larval and imaginal tissues. Evidence for these tissue-autonomous *Sxl/tra* growth inputs comes from previous studies [25,42] as well as our own finding that *Sxl* and *tra* activities in the larval fat body and wing imaginal disc contribute towards the increased size characteristic of these female tissues. We also found that Sxl and Tra were unable to increase fat body cell size in a cell-autonomous manner significantly in males or in females lacking neuronal Sxl. Hence, larval tissue-autonomous *Sxl/TraF* activity may only be able to boost growth efficiently in the presence of the Sxl-dependent signal from neurons. We therefore propose in our model that *Sxl* activity in neurons and in local tissues acts together to maximize female tissue growth.

### Parallels between the control of SSD in *Drosophila* and mammals

A widely held textbook view is that somatic sexual dimorphism is regulated very differently in *Drosophila* and mammals: the former in a cell-autonomous manner, the latter by gonad-derived hormones [13,14]. However, our discovery that the brain remotely regulates SSD in *Drosophila* may have its counterpart in mammals. Thus, mammalian gonadal sex steroids are thought to act on neurons in the hypothalamus to regulate growth hormone secretion from the anterior pituitary gland [reviewed in 5,29]. Male-female differences in the levels and patterns of circulating growth hormone are thought to induce sex-specific insulin-like growth factor 1 (IGF-1) profiles, in turn conferring dimorphic growth patterns [reviewed in 5,56]. Growth hormone itself does not appear to be conserved in *Drosophila*, but it is interesting that the pars intercerebralis of the *Drosophila* brain, harbouring the IPCs, has been likened to the mammalian hypothalamus [57]. Furthermore, IPCs send projections to the ring gland, which has been likened to the pituitary gland in mammals [57]. In a second parallel, our findings together with several studies in mammals [reviewed in 6,58] suggest that primary sex determination signals act via a combination of cell-autonomous and non-cell-autonomous mechanisms to control overall SSD. For example, in human and other mammalian embryos, sex differences in body size are noticeable before gonad differentiation, suggesting the existence of gonadal sex hormone–independent mechanisms of SSD [59,60]. Moreover, an XX rather than an XY chromosome complement in mice can increase adult body mass by approximately 7%, independently of gonadal sex, and this difference can be strongly enhanced by gonadectomy [61]. In conclusion, the upstream sex determination pathways in *Drosophila* and mammals are very different but the regulatory logic of how they regulate body growth via both cell-autonomous and remote mechanisms appears to be more similar than previously thought.

## Materials and methods

### Fly strains and culture

All stocks were maintained and experimental larvae grown on our standard (2× yeast) growth medium (58.5 g/L glucose, 6.63 g/L cornmeal, 23.4 g/L dried yeast, 7.02 g/L agar, 1.95 g/L Nipagen, 7.8 mg/L Bavistan). Where necessary to identify relevant larvae, stocks were balanced over Dfd::YFP- or GFP-marked balancers. The wild-type stock used in this study, including for control crosses for Gal4/UAS experiments, is *w*^*1118*^ *iso*^*31*^ (Wolbachia negative). For the growth curve in Fig 1A–1F, we used the offspring from a cross of *w*^*1118*^ *iso*^*31*^ females to males with an RFP-marked X chromosome (P-RFP, gift from A. Bailey) so that female progeny were fluorescently marked. Other fly stocks used in this study: UAS-Sxl RNAi 1 (TRiP.GL00634, Bloomington #38195), UAS-Sxl RNAi 2 (TRiP.HMS00609 Bloomington #34393), UAS-tra RNAi 1 (GD764, VDRC #2560, validated in [62]), UAS-tra RNAi 2 (TRiP.JF03132 Bloomington #28512, validated in [63]), UAS-tra2 RNAi 1 (GD768 VDRC #8868, also used in [25]), UAS-tra2 RNAi 2 (TRiP.HMS04334 Bloomington #56912), UAS-msl-2 RNAi (GD14745 VDRC #29356, validated in [34]), UAS-Sxl^alt5-8C^ (Bloomington #58484), UAS-TraF (Bloomington #4590, generated in [64]), UAS-Ilp2 [31], UAS-Ilp5 [31], UAS-p60 [65], lexAOP-Gal80 (Bloomington #32213), tsh-Gal80 [66] (made by Julie Simpson), elav^c155^-Gal4 (Bloomington #458), Cg-Gal4 (Bloomington #7011), r4-Gal4 (Bloomington #33832), Lpp-Gal4 [67], PromE(800)-Gal4 [36], Mex1-Gal4 [68], elav-Gal4^GMR71C07^ (Bloomington #46655), Insc-Gal4^MZ1407^ (Bloomington #8751), Gad1-Gal4 (Bloomington #51630), VGAT-Gal4 [69]), amon^386Y^-Gal4 (Bloomington #25410), dimm^c929^-Gal4 (Bloomington #25373), Ilp2-Gal4 [32], Ilp215-1-Gal4 and Ilp215-3-Gal4 [31], repo-Gal4 (Xiong, Mentall et al. 1994 G&D), elav^GMR27E06^-Gal4 (Bloomington #45530), ato-Gal4 (chordotonal organ, Bloomington #9494), Akh-Gal4 (corpora cardiaca, Bloomington #25683), Smid^c161^-Gal4 (peripheral nervous system, Bloomington #27893), ChAT-Gal4 (cholinergic, Bloomington #27893), D42-Gal4 (motorneurons, Bloomington #8816), Ddc-Gal4 (serotonergic/dopaminergic, Bloomington #7010), DMS-Gal4 (Drosomyosuppresin-neurons [mNSC subset], Bloomington #7010), Dsk-Gal4.P [41], Dsk-Gal4.TH (Bloomington #51981), Fru-Gal4.P1 [70], Fru-Gal4^NP0021^ (Bloomington #30027), GABA-B-R2-Gal4 [71], hugS3-Gal4 [72], MJ94-Gal4 (internal chemosensory neurons) [72], NPF-Gal4 (Bloomington # 25681), P0206-Gal4 (prothoracic gland and corpora allata) [73], Ple-Gal4 (dopaminergic, Bloomington #8848), Ppk-Gal4.G(2) and Ppk-Gal4.G(3) (peripheral nervous system, class IV multidendritic, Bloomington #32078 and #32079), sNFP-Gal4.TH (Bloomington #51991), sNPF-Gal4^NP6301^ (DGRC #113901), Tdc-Gal4 (octopaminergic, Bloomington #9313), Trh-Gal4 (serotonergic, Bloomington #38389), Vum-Gal4 (VUM neurons, Bloomington #29031), Ilp2-lexA::VP16 [74], Sxl^f7,M1^;; Sxl^+tCa^/+ (Bloomington #58486), Sxl^M1,fΔ33^/Binsinscy (Bloomington #58487), Ilp2^1^ [75], Df(Ilp1-5) [76], homozygous stock gift of R. Sousa-Nunes.

To label permanently cells expressing *elav*^*c155*^*-Gal4* with GFP, *elav*^*c155*^*-Gal4* flies were crossed to *act>stop>Gal4*, *UAS-GFP/GlaBc; UAS-Flp/TM6* (gift from Alberto Baena Lopez). To generate Flp out clones in the fat body, flies of the genotype *hs-Flp;; actin5C>CD2>Gal4*, *UAS-nls*::*GFP* were crossed to relevant UAS-lines and grown at 29°C from L1. This leads to low-frequency excision of the CD2 stop cassette in fat body clones, thus activating Gal4 and nls::GFP expression. *elav*^*c155*^-driven knockdown of either the *Sxl RNAi 1* or *Sxl RNAi 2* line abrogated larval body SSD. *Sxl RNAi 2* at 29°C caused a developmental delay to the wandering L3 stage (wL3) and female larvae failed to pupariate and/or eclose. This was not observed in *elav*^*c155*^*>Sxl RNAi 1* larvae, with females developing without noticeable delay and only showing lethality after adult eclosion. Late larval/pupal lethality in *elav*^*c155*^*>Sxl RNAi 2* females may be due to leaky RNAi expression, as poor fertility of the *UAS-Sxl RNAi 2* stock is observed even in the absence of Gal4, and the RNAi transgene appears to become silenced after prolonged maintenance of the stock at 25°C. For combined Sxl knockdown in *Ilp2-Gal4* and *Gad1-Gal4* cells, recombinant lines were generated with *Ilp2-Gal4*, *UAS-Sxl RNAi 1* and then crossed to *Gad1-Gal4* flies. Three independent recombinant lines were tested, giving similar results. To allow combination of *Ilp2>Sxl RNAi* with *Ilp2-lexA*::*VP16>lexOP-Gal80*, recombinant lines were generated with *Ilp2-lexA*::*VP16*, *UAS-Sxl RNAi 1*.

### Body mass measurements

Embryos were collected on grape juice-agar plates supplemented with yeast paste at 25°C. Larvae were then synchronised at L1 hatching and transferred onto 2× yeast food at fixed density. For all Gal4/UAS experiments, larvae were grown at 29°C until wL3 stage, washed in PBS, and weighed individually or in groups on a Satorious MSE3.6P microbalance. Ilp mutants were grown at 25°C until wL3. For the time course of larval growth, larvae were synchronised over 1–2 h at L1 hatching in batches at several intervals. All larvae were grown at 25°C and larvae were floated in 30% glycerol/PBS to measure body mass at relevant stages. For L1 larvae, males and females were transferred by pipetting onto droplets of PBS on grape juice-agar plates, and the liquid was then taken off and evaporated. Dry larvae were accumulated on the tip of a forceps, and the group of larvae was dropped into a weighing boat containing olive oil and resting on the tared microbalance. To promote adult survival of *elav*^*c155*^*>Sxl RNAi 1* females for experiments in Fig 6, experimental and control animals were reared at 18°C during pupal stages, resulting in approximately 20% survival for *elav*^*c155*^*>Sxl RNAi 1* females at 1–2 d posteclosion. Anaesthetized adults were weighed individually 1–2 d after eclosion on a Satorious MSE3.6P microbalance.

### *Gal4>Sxl RNAi* screen

Females of the genotype *UAS-Sxl RNAi 1/CyO*, *Dfd-YFP; UAS-Dcr2* were crossed to Gal4 driver males (except for Gal4 drivers located on the X chromosome, where Gal4 driver females were crossed to males of the genotype *UAS-Sxl RNAi 1/CyO*, *Dfd-YFP; UAS-Dcr2*). Larvae were grown and body mass was measured in groups as described above, after sorting larvae into *CyO*, *YFP*^+^ (no *UAS-Sxl RNAi* control group), and *CyO*, *YFP*^-^ (experimental group). Note that for some drivers that were balanced over *CyO*, *Dfd-YFP* (*Gad1-Gal4* and *Akh-Gal4*), the *CyO*, *YFP*^+^ group also included larvae carrying *UAS-Sxl RNAi* but no Gal4 driver.

### Food intake measurements

Larvae were synchronised at the L1/L2 or L2/L3 molt. To begin the food intake assay, groups of larvae were transferred by forceps directly from 2× yeast into 2× yeast + 1.5% erioglaucine disodium salt (SIGMA) within 3 minutes. After 20 minutes (L2 assay) or 25 minutes (L3 assay) from the first larva being transferred, larvae were floated in 30% glycerol, transferred into PBS, sorted into males and females, and weighed in groups before being ground up in PBS (20uL/mg of larval mass) using a tissue grinder and stored at -20C until quantification. As a blank control, larvae feeding on 2× yeast were processed in the same way. To generate a standard curve, 2× yeast + 1.5% erioglaucine disodium salt was dissolved in PBS at a known concentration (w/v) and a serial dilution was created. For quantification, all samples were centrifuged 1 minute at 12,000 g. Absorbance at 625 nm was measured in triplicate from the soluble fraction (avoiding top layer of lipid) using a NanoDrop 2000. Mean absorbance in the blank samples was subtracted from all experimental samples.

### Immunostaining and confocal microscopy

Larvae were inverted and fixed in 2% PFA in PBL (75 mM lysine, 37 mM sodium phosphate buffer [pH 7.4]) for 45 minutes at RT. For immunostainings against Ilp2 and dFoxo, larvae were removed from food one at a time, dissected, and transferred to PFA solution immediately. For anti-Ac-H4K16 stainings, larvae were fixed for 20 minutes in 4% PFA in PBS. After fixation, samples were washed in PBS and dissected further, if necessary. Samples were blocked in 10% normal goat serum (NGS) in PBS + 0.2% Triton (PBT). Primary antibodies and secondary antibodies were diluted in 10% NGS in PBT; washes were carried out in PBT. DAPI was added during primary or secondary antibody incubations. For FB stainings, the final wash was carried out overnight at 4°C to reduce DAPI cytoplasmic background staining. Primary antibodies used: rabbit anti-Ilp2 [33] at 1:800, rabbit anti-dFoxo [77] at 1:500, mouse anti-Sxl (M18 supernatant, Developmental Studies Hybridoma Bank) at 1:100, mouse anti-Elav (9F8A9 supernatant, Developmental Studies Hybridoma Bank) at 1:100, rabbit anti-Histone Ac-H4K16 (Active motif #39167) at 1:300, rabbit anti-Msl-2 (d-300) (Santa Cruz sc66969) at 1:50, rabbit anti-GABA (Sigma #A2052) at 1:1,000. All samples were mounted in Vectashield. For volume measurements, samples were mounted in a well generated by 1 or 2 layers of magic tape (Scotch) or a reinforcement ring (Avery) to avoid compression. All samples were imaged on a Leica SP5 upright microscope in oil. Samples for direct quantitative comparison were imaged on the same day using the same settings. Volume measurements were carried out using Volocity v6 software. Fat body nuclear diameters were measured manually using Fiji software, measuring mean diameter value of up to 10 nuclei per fat body. Ilp2 fluorescence intensity in IPCs was quantified on sum intensity projections of IPC clusters in Fiji software as the product of mean intensity x area of a manually selected region comprising the IPC cell bodies. Values for both IPC clusters were averaged for each brain. Quantification of Foxo nuclear and cytoplasmic localisation in the fat body was performed on Z-stacks of confocal images in Volocity v6 software. Nuclei were segmented using the DAPI channel followed by measurement of mean Foxo signal intensity in nuclei. Cytoplasm was segmented based on Foxo signal followed by subtraction of the nuclei, and mean Foxo signal intensity in the cytoplasm was determined. Ratio of mean nuclear to mean cytoplasmic Foxo signal was calculated for each fat body. A control experiment in which female larvae were starved for 4 h prior to fixation resulted in a 2.9-fold increase in the Foxo nuclear:cytoplasmic ratio, demonstrating the validity of our protocol.

### Western blotting

To prepare whole body lysates, groups of larvae were homogenised in cold lysis buffer (50 mM Tris pH 7.5, 250 mM NaCl, 5 mM EDTA, 0.5% NP-40, 50 mM NaF) + protease/phosphatase inhibitor tablet (Pierce #88669) at a ratio of 20 μL per mg of larval material. Homogenates were centrifuged for 15 minutes at 4°C to pellet insoluble material. The supernatant was resuspended in Laemmli sample buffer and boiled for 3 min at 95°C. Proteins were separated on 4%–15% NuPage gels (Invitron) in MOPS under reducing conditions and transferred onto nitrocellulose using standard procedures. Primary antibodies (mouse anti-tubulin [abcam ab44928]), rabbit anti-Akt (Cell Signalling #4691S), and rabbit anti-pAkt (Cell Signalling #4054S) were detected with fluorescent secondary antibodies (LI-COR) on a LI-COR Odyssey scanner. Bands were quantified from the raw image using Image Studio Lite.

### qPCR

Groups of 1–3 h L3 larvae were homogenized in 500 μL TRIzol (Invitrogen #15596018) and RNA was extracted according to manufacturer’s protocol. RNA extracts were treated with RQ1 RNase-free DNAse I (Promega #M6101) according to manufacturer’s protocol to remove any genomic DNA contaminants. cDNA was prepared using the SuperScript IV First Strand cDNA Synthesis system (Thermo Fisher Scientific #18091200) with 1 μg total RNA and using oligo(dT)_20_ primers, following the kit protocol with an annealing temperature of 52.5°C. qPCR was performed on a Roche LightCycler 480 II using the LightCycler 480 SYBR Green I Master mix (Roche #18887320), 500 nM final primer concentration, and 1:25 final dilution of cDNA. The following qPCR primers were used:

dmyc [43]: 5′-GACGGATACGGAAACTATGT and 5′-GTAAAGGGCCATTGCGATTA
tubulin: 5′-TGTCGCGTGTGAAACACTTC and 5′-AGCAGGCGTTTCCAATCTG
RNApol2: 5′-CCTTCAGGAGTACGGCTATCATC and 5′-CCAGGAAGACCTGAGCATTAATCT

For dmyc primers, qPCR reactions had an annealing temperature of 65°C; for other primers, the annealing temperature was 60°C.

## Supporting information

S1 Fig Mid-L2 and early-L3 larvae show no sex differences in insulin signalling. **(A-B)** Immunostaining for Ilp2 levels in insulin producing cells (IPCs) in mid-L2 (A) and early L3 (B) larvae. Ilp2 quantifications show mean, SD and individual data points for each CNS (see Materials and methods). **C)** Western blot for phospho-Akt, Akt and tubulin in whole body lysates of early L3 larvae. Quantification shows mean, SD and individual replicates.**(D)** Immunostaining for FoxO in fat bodies of mid-L2 and early L3 larvae. Quantification shows ratio of mean signal intensity of FoxO in nucleus vs. cytoplasm (see Materials and methods), plotted as mean, SD and individual data points show ratios for each larva. No significant differences (p<0.05) were detected between the sexes for any of the three readouts of insulin signalling, according to unpaired t-tests. Scale bar in (A-B) 10μm, in (D) 10μm.
(TIF)

S2 Fig Male and female larvae differ in absolute but not mass-specific food intake. Absolute (per larva) and mass-specific (per mg of larval body mass) food intake in early L2 and early L3 larvae, measured during 20 min (early L2) or 25 min (early L3) in groups of 9–20 larvae. Graphs plot mean, SD and data points for individual replicates. P-values are shown according to paired t-tests.
(TIF)

S3 Fig *Sxl*, *tra* and tra-2 manipulations in the fat body have minor effects on female body size and SSD. **(A-B)** Body mass of wandering L3 larvae expressing *tra* RNAi in the fat body using Cg-Gal4. (A) shows mean body mass per larva in mg (weighed in groups of n = 6–14 larvae) and (B) shows mean body mass and SD of individually weighed larvae (n = 27–30 larvae per group). **(C)** Body mass of wandering L3 larvae expressing *tra* or *tra-2* RNAi in the fat body using *Lpp-Gal4*. Graph shows mean body mass (mg), SD and individual data points. **(D-F)** Body mass of wandering L3 larvae expressing *Sxl* RNAi in the fat body using the Gal4 drivers *Cg-Gal4* (D), *Lpp-Gal4* (E) or *r4-Gal4* (F). (D) shows mean body mass and SD of 2–3 replicates of groups of 2–12 larvae; (E) shows mean body mass, SD and individual measurements; (F) shows mean body mass and SD for groups of individually weighed larvae (n = 4–11 larvae per group).
(TIF)

S4 Fig *Sxl* knockdown in oenocytes or midgut does not affect female body size and SSD. **(A)** Body mass of wandering L3 larvae expressing *Sxl* RNAi in oenocytes using *PromE-Gal4*. Graph shows mean, SD and individual data points. **(B)** Body mass of wandering L3 larvae expressing *Sxl* RNAi in midgut enterocytes using *Mex1-Gal4*. Graph shows mean body mass and SD for n = 3–16 replicates of groups of larvae (5–11 larvae per group).
(TIF)

S5 Fig Neuronal knockdown of *Sxl* abolishes the establishment of larval body SSD. **(A)** Body mass of control and *elav^c155^>Sxl RNAi 1* larvae at 3-24h after synchronization at the L1/L2 molt. Early L3 occurs at ~24h after L1/L2 molt. Mean body mass and SD was measured for replicate groups of 3–6 larvae (3h, 6h and 11h time points) or for individual larvae (18h and 24h time points). Control larvae show statistically significant (p<0.05) sex differences in body mass from 6h after the L1/L2 molt but *elav^c155^>Sxl RNAi 1* larvae show no significant sex difference in body mass at any time point. (B) Female to male (F:M) body mass ratio (SSD) and SEM for control and *elav^c155^>Sxl RNAi* larvae calculated from the data in (A). Neuronal Sxl is required for the establishment and maintenance of larval body SSD during L2.
(TIF)

S6 Fig Loss of neuronal Sxl expression following knockdown of *Sxl* and *msl-2* or *Sxl* alone. (A) Sxl immunostaining in the early L3 thoracic ventral nerve cord of control, *elav^c155^>Sxl RNAi* and and *elav^c155^>Sxl RNAi + msl-2 RNAi* larvae. DNA is labelled by DAPI. **(B)** Quantification of Sxl expression (mean signal intensity) in regions of interest similar to those shown in (A). Neuronal knockdown with Sxl RNAi 1 efficiently blocks Sxl expression and this is not rescued by additional knockdown with msl-2 RNAi. The very low levels of Sxl in males likely represent non-specific background staining.
(TIF)

S7 Fig Sxl does not function in glia to control larval body SSD. **(A)** Confocal image projection of a late third instar larval CNS, with *elav^c155^-Gal4*-expressing cells permanently marked with GFP (green) and glial cells stained by anti-Repo antibody (red). There is no detectable overlap between *elav^c155^-Gal4* driver activity and Repo signal. **(B)** Confocal image projections of CNSs from female L3 larvae, immunostained with anti-Sxl antibody. In the control CNS, Sxl shows a broad expression throughout the CNS. Knockdown of Sxl with *elav^c155^>Sxl RNAi* results in residual Sxl staining with a distribution resembling that seen with Repo (see A), suggesting that Sxl expression in glia is not affected. Note that Sxl expression is also preserved in the prothoracic gland of the ring gland (white arrow), which does not express *elav^c155^-Gal4*. Scale bars in A and B: 50μm. **(C)** SSD is normal in larvae expressing *Sxl* RNAi in glial cells using *Repo-Gal4*. Left graph shows mean body mass, SD and individual data points. Right graph shows mean and SEM for female to male (F:M) ratio of larval body mass, a measure of SSD.
(TIF)

S8 Fig SSD screen of Gal4 drivers using *UAS-Sxl RNAi*. Females of the genotype *UAS-Sxl RNAi 1/CyO*, *Dfd-YFP; UAS-Dcr2* were crossed to control or Gal4 driver males but, for Gal4 drivers located on the X-chromosome, the cross was reversed. Wandering L3 larvae were sorted by sex and YFP expression into *UAS-Sxl RNAi 1*versus *CyO*, *Dfd-YFP* genotypes. Mean body masses and SDs for data for replicate groups are shown. Hits were identified by manual inspection as Gal4-drivers that decreased the body size of females but not males, relative to their CyO balancer controls. The presence of the *CyO, Dfd-YFP^+^* balancer did not significantly affect larval body size, as seen in the control cross without a Gal4 driver.
(TIF)

S9 Fig Expression patterns of the Gad1-GAL4 and VGAT-GAL4 drivers in this study. Confocal Z-projections of the CNSs from late L3 larvae showing Gad1-GAL4 (A) or VGAT-GAL4 (B) driving expression of membrane-targeted GFP (UAS-CD8::GFP) in green. DNA is stained by DAPI in blue.
(TIF)

S10 Fig Expression patterns of Ilp2-Gal4 and Ilp2-lexA drivers used in this study. Confocal Z-projections of the CNSs from female late L3 larvae showing expression of membrane-targeted GFP (UAS-CD8::GFP or lexAOP-CD2::GFP) in green and DNA stained by DAPI in blue. Ilp2-Gal4 and Ilp215-1-Gal4 are weakly active in some neurons of the ventral nerve cord (VNC) and their projections (white arrows). Ilp2-lexA::VP16 shows no detectable expression in the VNC.
(TIF)

S11 Fig Restoration of Sxl in IPCs rescues the SSD phenotype of Ilp2>Sxl RNAi larvae. (A-C) Single confocal sections through clusters of IPCs from female larvae, immunostained for Sxl and Ilp2 (to mark IPCs), with DNA stained by DAPI (blue). Sxl in the nuclei of IPCs is strongly expressed in control females (A) but absent in those of Ilp2-Gal4>UAS-Sxl RNAi 1 larvae (B). Driving Gal80 (which blocks Gal4 activity) specifically in the IPCs of Ilp2-Gal4>Sxl RNAi 1 female larvae using the LexAOP system (Ilp2-lexA::VP16>lexAOP-Gal80) restores Sxl expression in the IPCs either fully (C) or partially (C’). Scale bar: 10mm. (D) Sxl expression is restored in all seven IPCs of ~80% of the female larvae carrying Ilp2-Gal4>UAS-Sxl RNAi + Ilp2-lexA::VP16>lexAOP-Gal80 (same genotype as C). (E) Restoration of Sxl expression in the IPCs of Ilp2>Sxl RNAi larvae (same genotype as in C and D) rescues the female to male (F:M) body mass ratio (SSD) of wandering L3 larvae. The underlying data for this figure can be found in S1 Data.
(TIF)

S12 Fig SSD phenotypes of Sxl knockdown with Ilp215-1-Gal4, VGAT-Gal4 or amon386Y-Gal4 are not altered by blocking Gal4 activity in VNC neurons with tsh-Gal80. (A) CNS expression patterns in female larvae at late L3 of Ilp215-1-Gal4, VGAT-Gal4 or amon386Y-Gal4 driving UAS-CD8::GFP (green) in the absence (left) or presence (right) of tsh-Gal80. All images show confocal Z-projections with DNA stained by DAPI (blue). tsh-Gal80 efficiently supresses Gal4 activity in ventral nerve cord (VNC) neurons and their projections, including those in which Ilp215-1-Gal4 is expressed (white arrow and also see S9 Fig). (B) tsh-Gal80 does not suppress the decrease in larval body SSD resulting from Ilp215-1-Gal4, VGAT-Gal4 or amon386Y-Gal4 driving UAS-Sxl RNAi 1. Graph plots the mean and SEM of female to male (F:M) body mass ratios of wandering L3 larvae with individual data points representing mean F:M ratios from 3–4 independent experiments. **** indicates p<0.0001 and ns that p>0.05 using one-way ANOVA with multiple comparisons. The underlying data for this figure can be found in S1 Data.
(TIF)

S13 Fig IPC and Ilp manipulations affecting Insulin signalling do not alter larval body SSD. (A) IPC overexpression of UAS-Ilp2 or UAS-Ilp5 using a combination of Ilp2-GAL4 + Ilp215-3-Gal4 has no effect on body size or SSD. (B) IPC inhibition of PI3K signalling (UAS-p60 driven by Ilp2-GAL4 + Ilp215-3-Gal4) reduces body size in both males and females but it does not decrease larval body SSD. (C) IPC inhibition of PI3K signalling (UAS-p60 driven by Ilp2-GAL4 + Ilp215-3-Gal4) reduces the female size and SSD of wing imaginal discs. (D) Ilp2 null mutant (Ilp21) and Ilp1,2,3,4,5-deficient (Df(Ilp1-5)) larvae have decreased male and female body mass but no change in larval body SSD. Data for all graphs were obtained from wandering L3 larvae. Histograms show means, SD and individual data points. Mean female to male ratios (F:M ratios) and SEMs refer as indicated either to body mass or to wing disc volume. * indicates p<0.05 and **** indicates p<0.0001 using one-way ANOVA with multiple comparisons. The underlying data for this figure can be found in S1 Data.
(TIF)

S14 Fig amon386Y-Gal4 and VGAT-Gal4 but not Gad1-Gal4 are expressed in IPCs. Confocal Z-projections of CNSs from late L3 larvae. UAS-CD8::GFP expression reveals that amon386Y-Gal4 (A) and VGAT-Gal4 (B) but not Gad1-Gal4 (C) are expressed in IPCs (marked by anti-Ilp2 immunostaining, Ilp2 also marks IPC projections in the corpora cardiaca of the ring gland, RG). Low power views of the CNS (left panels, scale bar 50mm) and higher magnifications of the IPCs (right panels, corresponding to boxed region in left panels) are shown. The signal intensity of GAL4 driver expression (green) has been increased in the right panels.
(TIF)

S15 Fig Cell-autonomous SSD contributions of Sxl and tra in the wing imaginal disc and larval fat body. (A-B) nubbin-Gal4 driving expression of UAS-Sxl RNAi 1, UAS-tra RNAi 1, UAS-TraF or UAS-InR RNAi decreases the SSD of the wing pouch. Gal4-expressing cells in the wing pouch were marked by UAS-RFP. To measure cell-autonomous effects, the mean ratios and SEMs of the volumes of the wing pouch (expressing nubbin-Gal4) were normalised to the volumes of the whole wing disc (A), although normalization to only the non-pouch region of the disc gives similar results. Note that Sxl and Tra depletion specifically reduce female wing pouch volume, whereas TraF overexpression specifically increases male wing pouch volume. Insulin receptor (InR) knockdown reduces wing pouch sizes in both males and females but the effect is stronger in females. The corresponding mean and SEMs for female to male (F:M) ratios of wing pouch volumes normalised to whole wing disc volumes are also shown (B). Note that for this type of F:M ratio, control genotypes have a value of ~1.0 and that the values of less than 1.0 seen with all three genetic manipulations indicate decreased wing pouch SSD. (C) Decreased nuclear diameter of fat body cells expressing UAS-Sxl RNAi 1 or UAS-tra RNAi 1 but not UAS-TraF. Fat body clones were generated with hsFlp; actin5C>CD2>Gal4 and marked with UAS-nls::GFP. Graph plots the mean ratios of the nuclear diameter (a proxy for cell size) of GFP+/GFP- fat body cells. Each data point shows the mean ratio from one clone and error bars indicate the SD. Sxl and Tra knockdown decrease nuclear diameter in the fat body of females, whereas TraF overexpression has no effect on nuclear diameter in the fat body of either sex. The underlying data for this figure can be found in S1 Data.
(TIF)

S16 Fig Female-biased expression of dmyc is maintained in elavc155>Sxl RNAi larvae. Graph shows qPCR quantitation of dmyc transcript levels from whole early L3 larvae, normalised to the geometric mean of tubulin and RNA polymerase II transcript levels. Individual data points are shown for replicates normalised to the mean female value for each genotype and pooled from two independent experiments (circles and triangles). Control larvae express dmyc at levels ~1.6 fold higher in females than males and this difference is retained in elavc155>Sxl RNAi larvae despite their loss of larval body SSD (see Fig 3A). ** indicates p<0.01 and *** shows p<0.001 using one-way ANOVA with multiple comparisons. The underlying data for this figure can be found in S1 Data.
(TIF)

S1 Table Expression patterns of Gal4 drivers. Expression patterns were examined in late L3 larvae, with Gal4 drivers activating the expression of *UAS-CD8*::*GFP*. For *elav^c155^-Gal4*, expression was examined using *act>stop>Gal4*, *UAS-GFP/GlaBc; UAS-Flp/TM6* to permanently label Gal4-expressing cells. An X or listed structures indicates expression present in some or all cells of this tissue/structure. Abbreviations: CC, corpora cardiaca; CNS, central nervous system; ECs, enterocytes; EEs, enteroendocrine cells; mNSCs, median neurosecretory cells; PNS, peripheral nervous system; SG, salivary gland; SNS, somatogastric nervous system; VNC, ventral nerve cord. “GABAergic” refers to the observation that expression of these Gal4 drivers only overlaps partially with GABA+ neurons in the early L3 female CNS (see S1 and S2 Images).
(DOCX)

S1 Data Underlying data for main and supporting figures. In this Excel file, separate worksheets contain the data used in each figure panel as indicated.
(XLSX)

S1 Image Partial overlap of GABA and *Gad1-Gal4* expression in early L3 female CNS. Confocal z-stack of early third instar female CNS, with *Gad1-Gal4* driving UAS-CD8::GFP (green), immunostaining for GABA (red) and DAPI staining (blue). This Tiff file can be viewed in ImageJ.
(ZIP)

S2 Image Partial overlap of GABA and *VGAT-Gal4* expression in early L3 female CNS. Confocal z-stack of early third instar female CNS, with *VGAT-Gal4* driving UAS-CD8::GFP (green), immunostaining for GABA (red) and DAPI staining (blue). This Tiff file can be viewed in ImageJ.
(ZIP)

## Abbreviations

ALH: after larval hatching
CNS: central nervous system
CW: critical weight
FoxO: forkhead box, sub-group O
H4K16: histone H4 lys16
IGF: insulin-like growth factor
Ilp: insulin-like peptide
InR: insulin receptor
IPC: insulin-producing cell
*msl-2*: *male-specific lethal 2*
SSD: sexual size dimorphism
*Sxl*: *Sex-lethal*
Tor: target of rapamycin
*tra*: *transformer*
*TraF*: the female-specific *tra* splice variant
UAS: upstream activation sequence
VNC: ventral nerve cord
