# Supplementary figures and images for: The sex of specific neurons controls female body growth in Drosophila
Source: PLoS Biol. 2017 Oct 4;15(10):e2002252. doi: 10.1371/journal.pbio.2002252 (PMC5627897; doi:10.1371/journal.pbio.2002252)

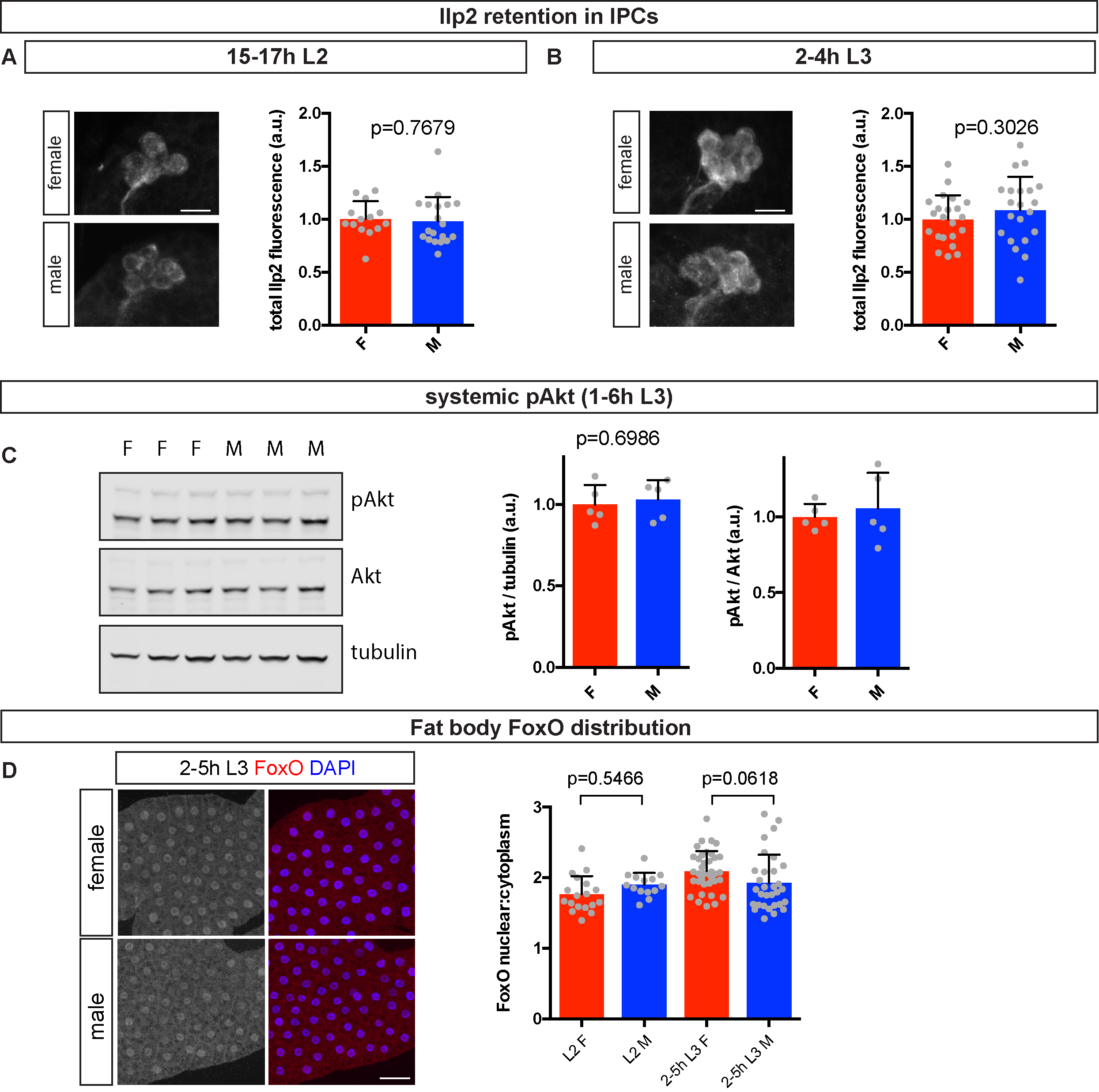

Supplement: S1 Fig — (A-B) Immunostaining for Ilp2 levels in insulin producing cells (IPCs) in mid-L2 (A) and early L3 (B) larvae. Ilp2 quantifications show mean, SD and individual data points for each CNS (see Materials and methods). C) Western blot for phospho-Akt, Akt and tubulin in whole body lysates of early L3 larvae. Quantification shows mean, SD and individual replicates.(D) Immunostaining for FoxO in fat bodies of mid-L2 and early L3 larvae. Quantification shows ratio of mean signal intensity of FoxO in nucleus vs. cytoplasm (see Materials and methods), plotted as mean, SD and individual data points show ratios for each larva. No significant differences (p<0.05) were detected between the sexes for any of the three readouts of insulin signalling, according to unpaired t-tests. Scale bar in (A-B) 10μm, in (D) 10μm. (TIF) [file pbio.2002252.s001.tif]

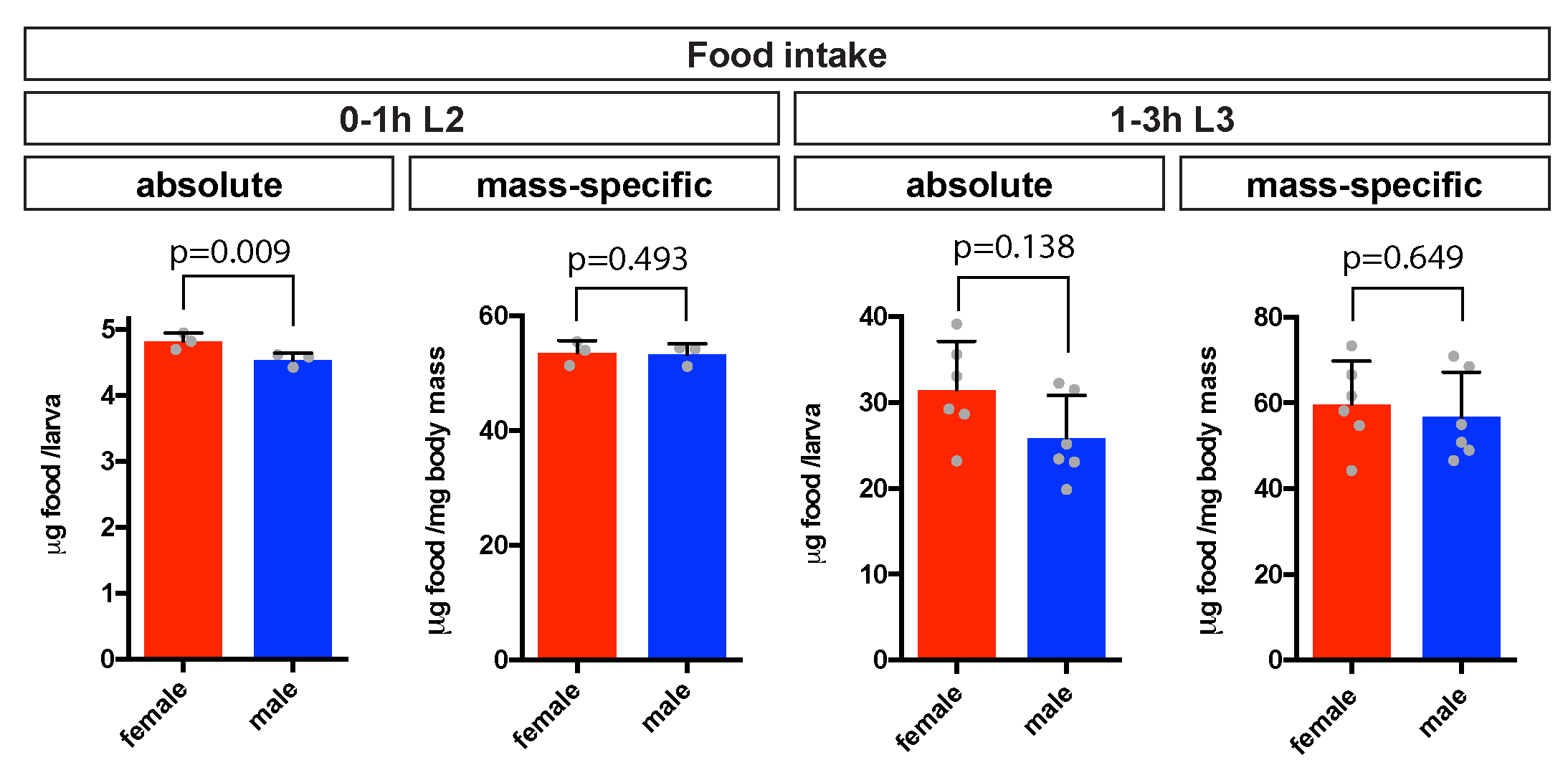

Supplement: S2 Fig — Absolute (per larva) and mass-specific (per mg of larval body mass) food intake in early L2 and early L3 larvae, measured during 20 min (early L2) or 25 min (early L3) in groups of 9–20 larvae. Graphs plot mean, SD and data points for individual replicates. P-values are shown according to paired t-tests. (TIF) [file pbio.2002252.s002.tif]

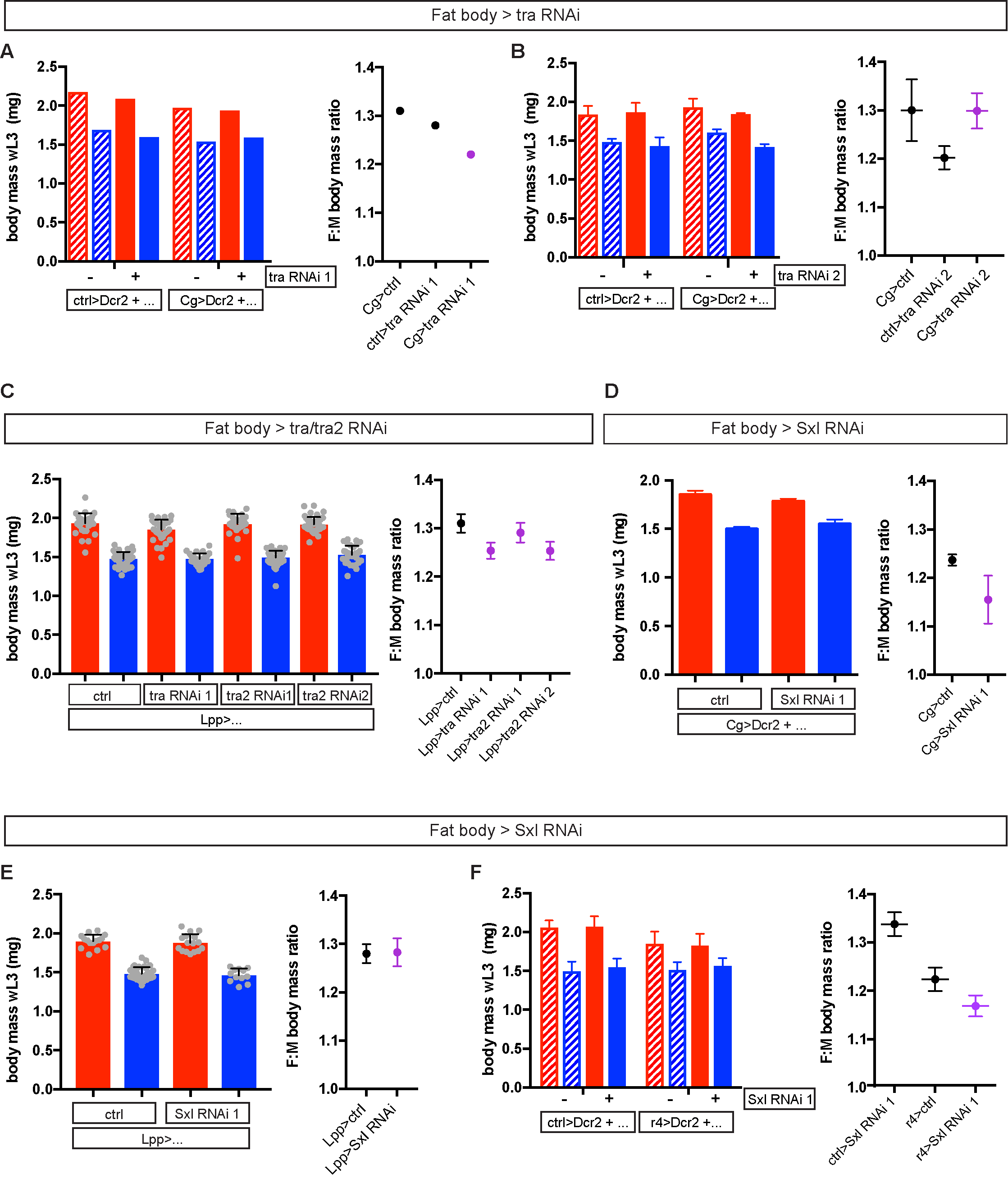

Supplement: S3 Fig — (A-B) Body mass of wandering L3 larvae expressing tra RNAi in the fat body using Cg-Gal4. (A) shows mean body mass per larva in mg (weighed in groups of n = 6–14 larvae) and (B) shows mean body mass and SD of individually weighed larvae (n = 27–30 larvae per group). (C) Body mass of wandering L3 larvae expressing tra or tra-2 RNAi in the fat body using Lpp-Gal4. Graph shows mean body mass (mg), SD and individual data points. (D-F) Body mass of wandering L3 larvae expressing Sxl RNAi in the fat body using the Gal4 drivers Cg-Gal4 (D), Lpp-Gal4 (E) or r4-Gal4 (F). (D) shows mean body mass and SD of 2–3 replicates of groups of 2–12 larvae; (E) shows mean body mass, SD and individual measurements; (F) shows mean body mass and SD for groups of individually weighed larvae (n = 4–11 larvae per group). (TIF) [file pbio.2002252.s003.tif]

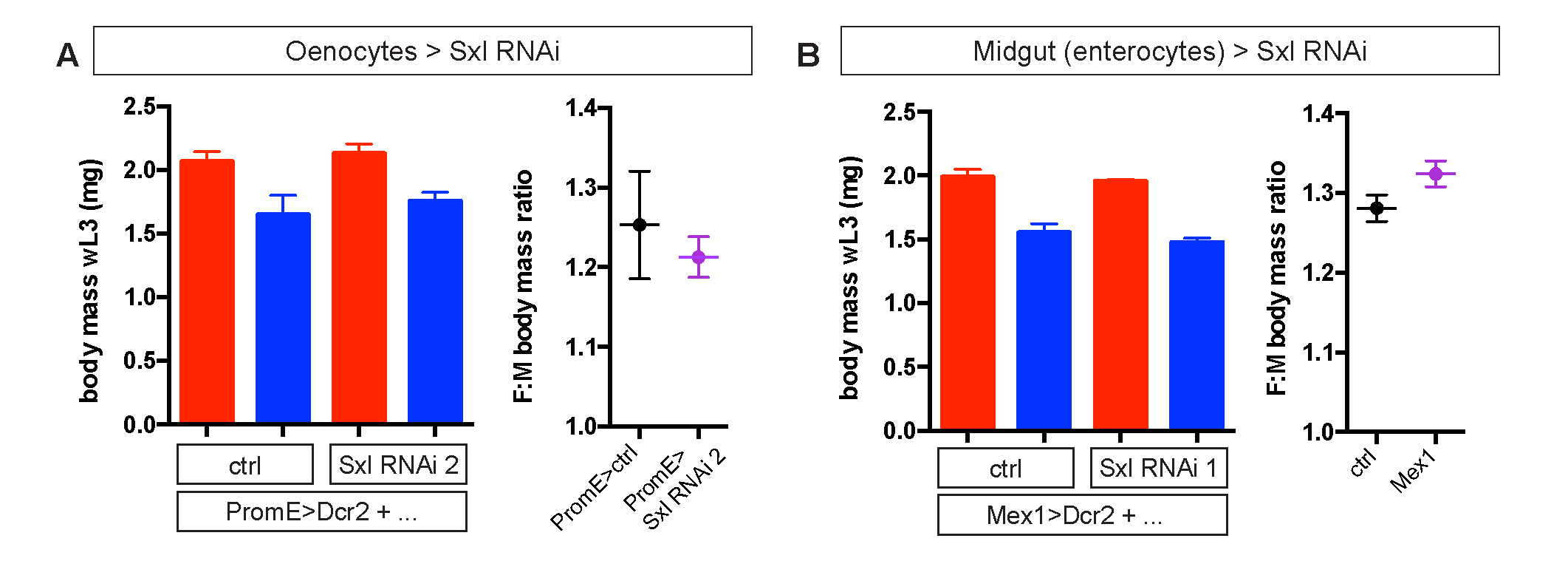

Supplement: S4 Fig — (A) Body mass of wandering L3 larvae expressing Sxl RNAi in oenocytes using PromE-Gal4. Graph shows mean, SD and individual data points. (B) Body mass of wandering L3 larvae expressing Sxl RNAi in midgut enterocytes using Mex1-Gal4. Graph shows mean body mass and SD for n = 3–16 replicates of groups of larvae (5–11 larvae per group). (TIF) [file pbio.2002252.s004.tif]

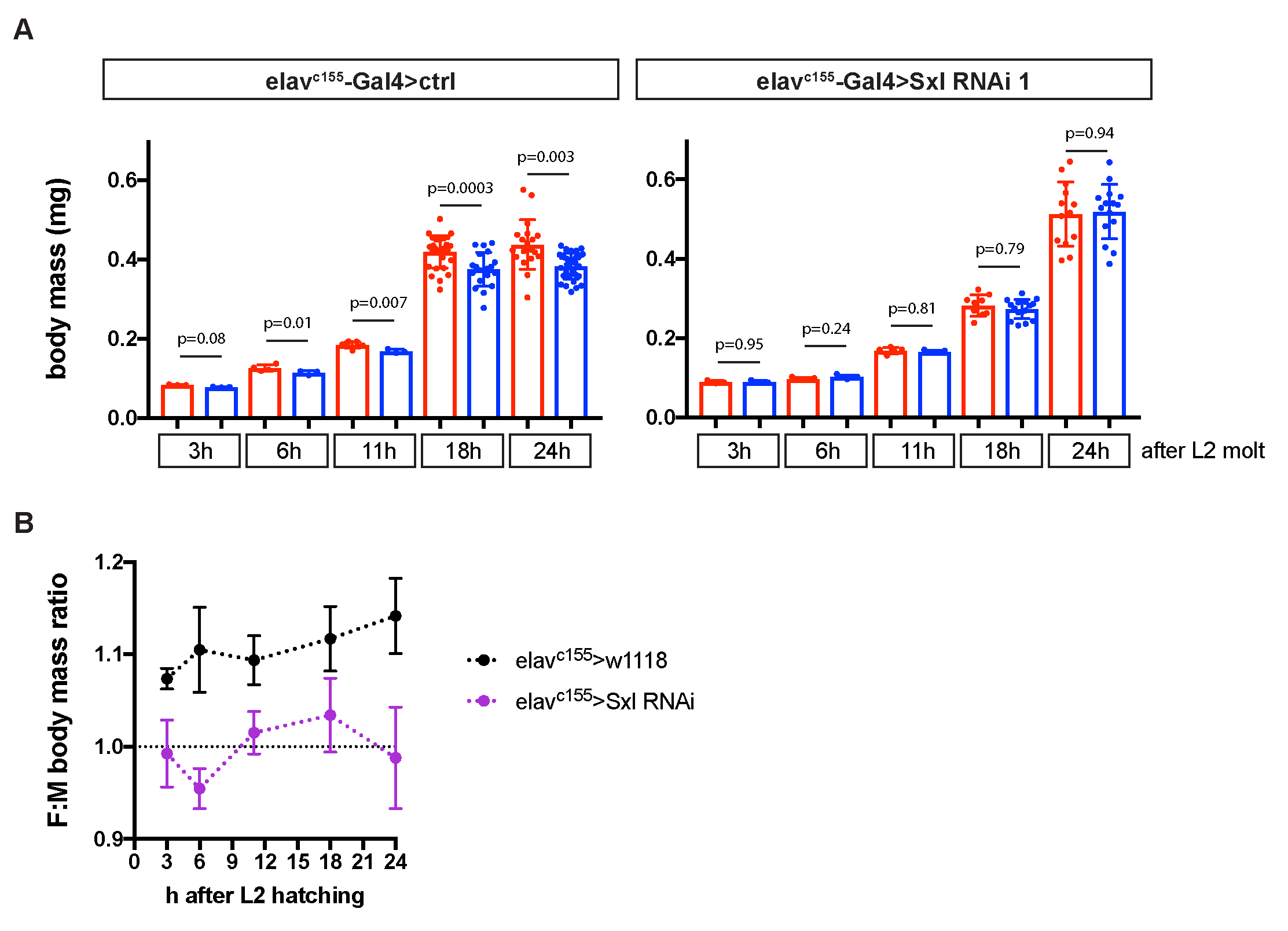

Supplement: S5 Fig — (A) Body mass of control and elavc155>Sxl RNAi 1 larvae at 3-24h after synchronization at the L1/L2 molt. Early L3 occurs at ~24h after L1/L2 molt. Mean body mass and SD was measured for replicate groups of 3–6 larvae (3h, 6h and 11h time points) or for individual larvae (18h and 24h time points). Control larvae show statistically significant (p<0.05) sex differences in body mass from 6h after the L1/L2 molt but elavc155>Sxl RNAi 1 larvae show no significant sex difference in body mass at any time point. (B) Female to male (F:M) body mass ratio (SSD) and SEM for control and elavc155>Sxl RNAi larvae calculated from the data in (A). Neuronal Sxl is required for the establishment and maintenance of larval body SSD during L2. (TIF) [file pbio.2002252.s005.tif]

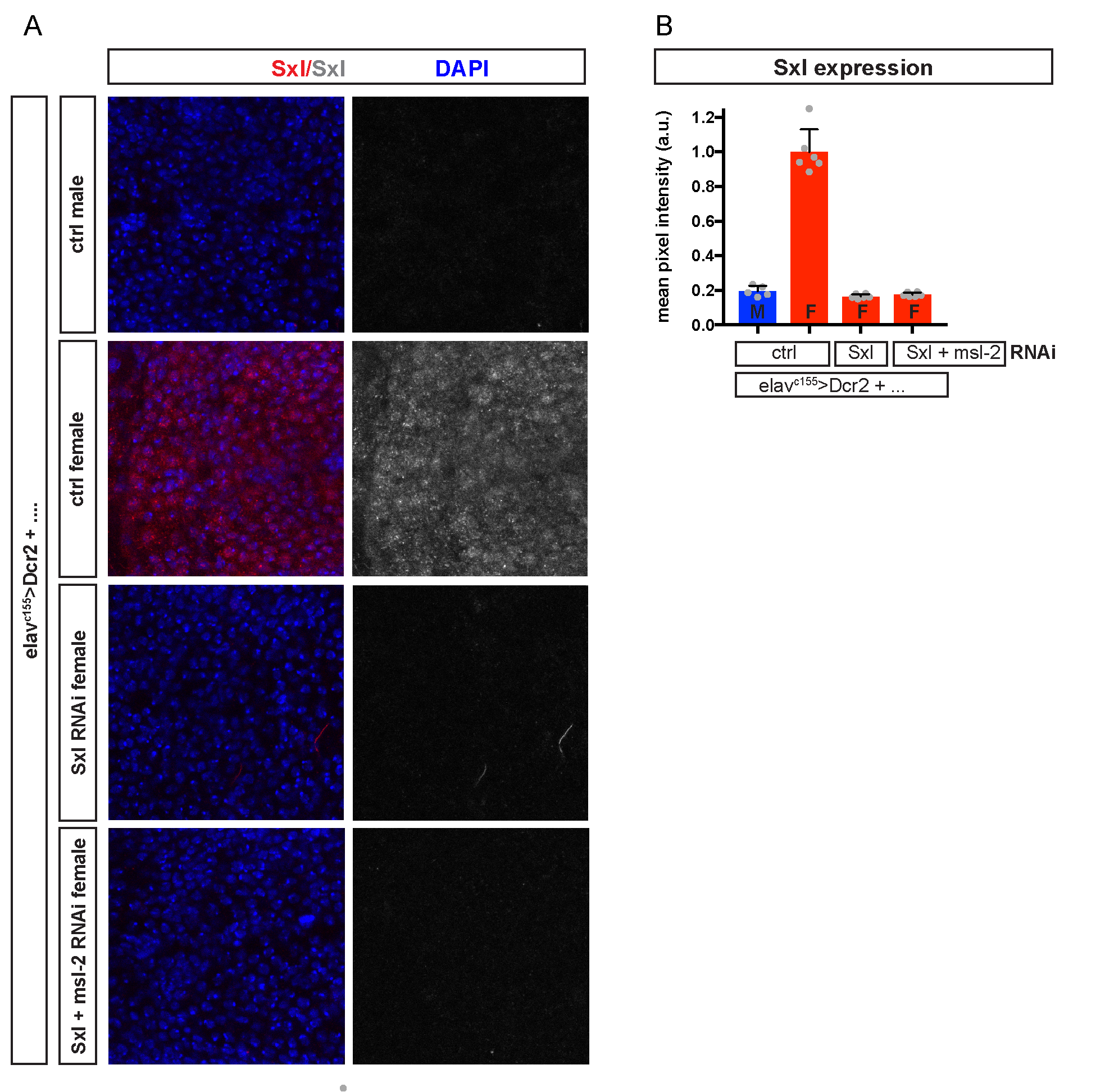

Supplement: S6 Fig — (A) Sxl immunostaining in the early L3 thoracic ventral nerve cord of control, elavc155>Sxl RNAi and and elavc155>Sxl RNAi + msl-2 RNAi larvae. DNA is labelled by DAPI. (B) Quantification of Sxl expression (mean signal intensity) in regions of interest similar to those shown in (A). Neuronal knockdown with Sxl RNAi 1 efficiently blocks Sxl expression and this is not rescued by additional knockdown with msl-2 RNAi. The very low levels of Sxl in males likely represent non-specific background staining. (TIF) [file pbio.2002252.s006.tif]

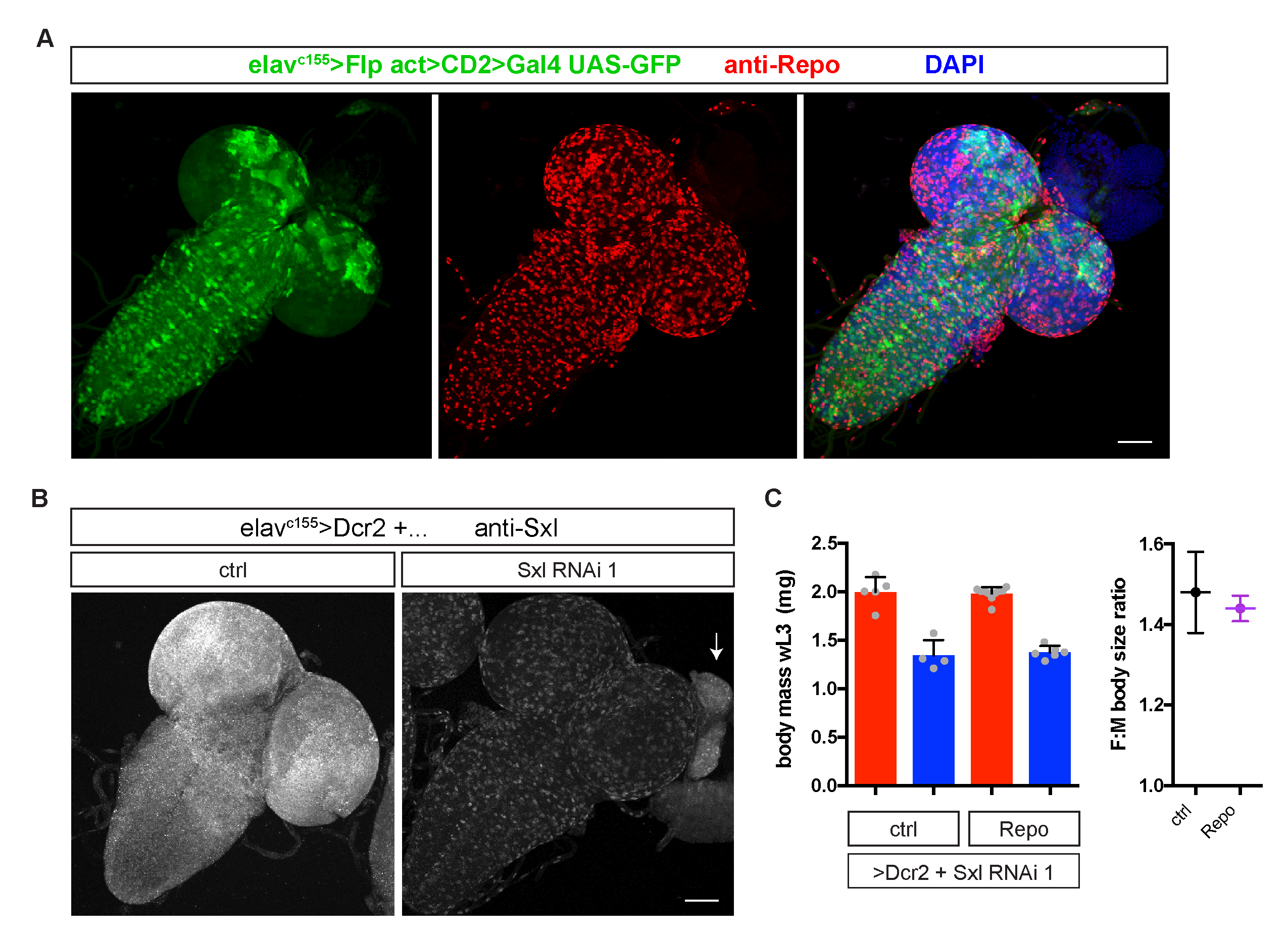

Supplement: S7 Fig — (A) Confocal image projection of a late third instar larval CNS, with elavc155-Gal4-expressing cells permanently marked with GFP (green) and glial cells stained by anti-Repo antibody (red). There is no detectable overlap between elavc155-Gal4 driver activity and Repo signal. (B) Confocal image projections of CNSs from female L3 larvae, immunostained with anti-Sxl antibody. In the control CNS, Sxl shows a broad expression throughout the CNS. Knockdown of Sxl with elavc155>Sxl RNAi results in residual Sxl staining with a distribution resembling that seen with Repo (see A), suggesting that Sxl expression in glia is not affected. Note that Sxl expression is also preserved in the prothoracic gland of the ring gland (white arrow), which does not express elavc155-Gal4. Scale bars in A and B: 50μm. (C) SSD is normal in larvae expressing Sxl RNAi in glial cells using Repo-Gal4. Left graph shows mean body mass, SD and individual data points. Right graph shows mean and SEM for female to male (F:M) ratio of larval body mass, a measure of SSD. (TIF) [file pbio.2002252.s007.tif]

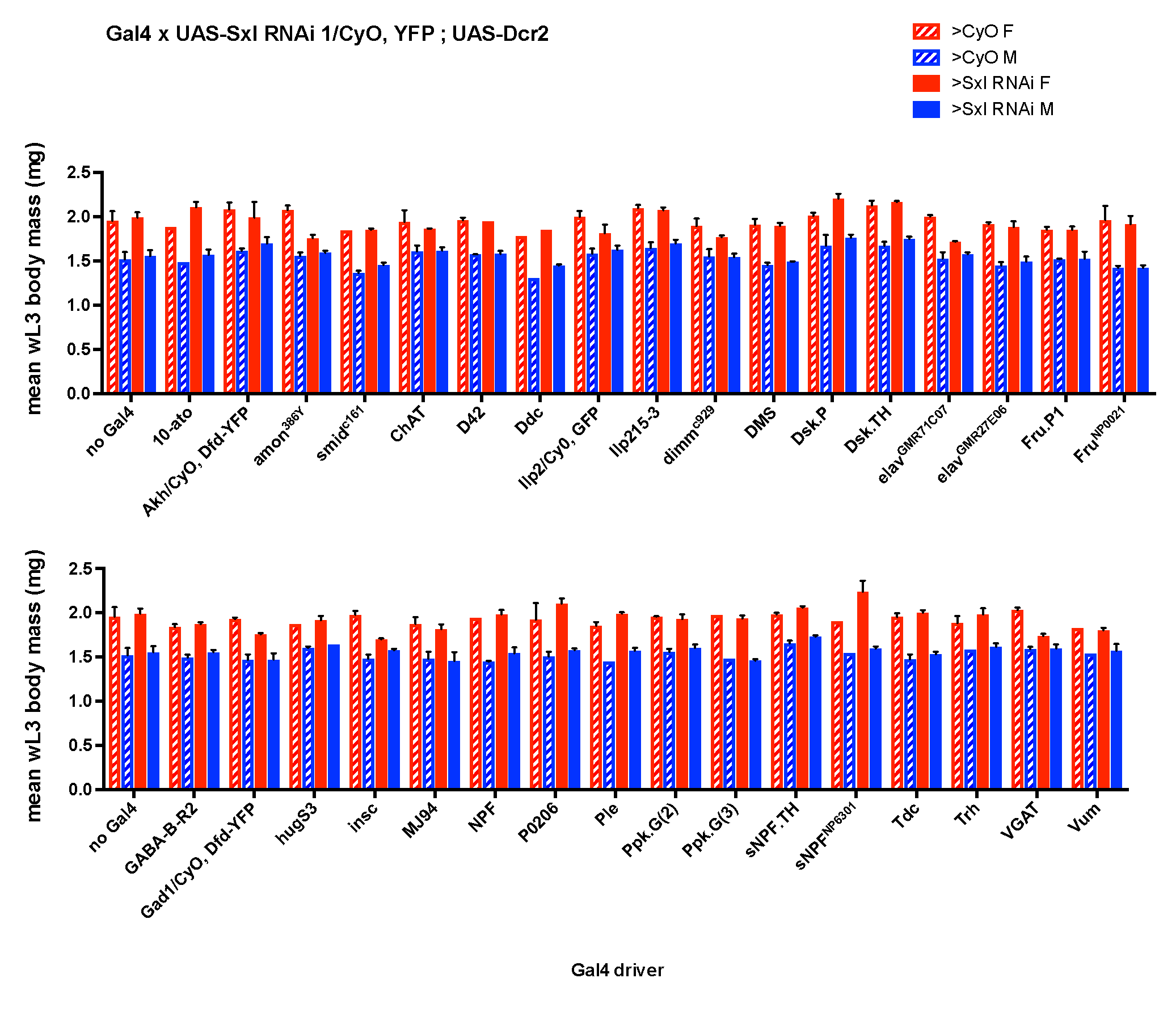

Supplement: S8 Fig — Females of the genotype UAS-Sxl RNAi 1/CyO, Dfd-YFP; UAS-Dcr2 were crossed to control or Gal4 driver males but, for Gal4 drivers located on the X-chromosome, the cross was reversed. Wandering L3 larvae were sorted by sex and YFP expression into UAS-Sxl RNAi 1versus CyO, Dfd-YFP genotypes. Mean body masses and SDs for data for replicate groups are shown. Hits were identified by manual inspection as Gal4-drivers that decreased the body size of females but not males, relative to their CyO balancer controls. The presence of the CyO, Dfd-YFP+ balancer did not significantly affect larval body size, as seen in the control cross without a Gal4 driver. (TIF) [file pbio.2002252.s008.tif]

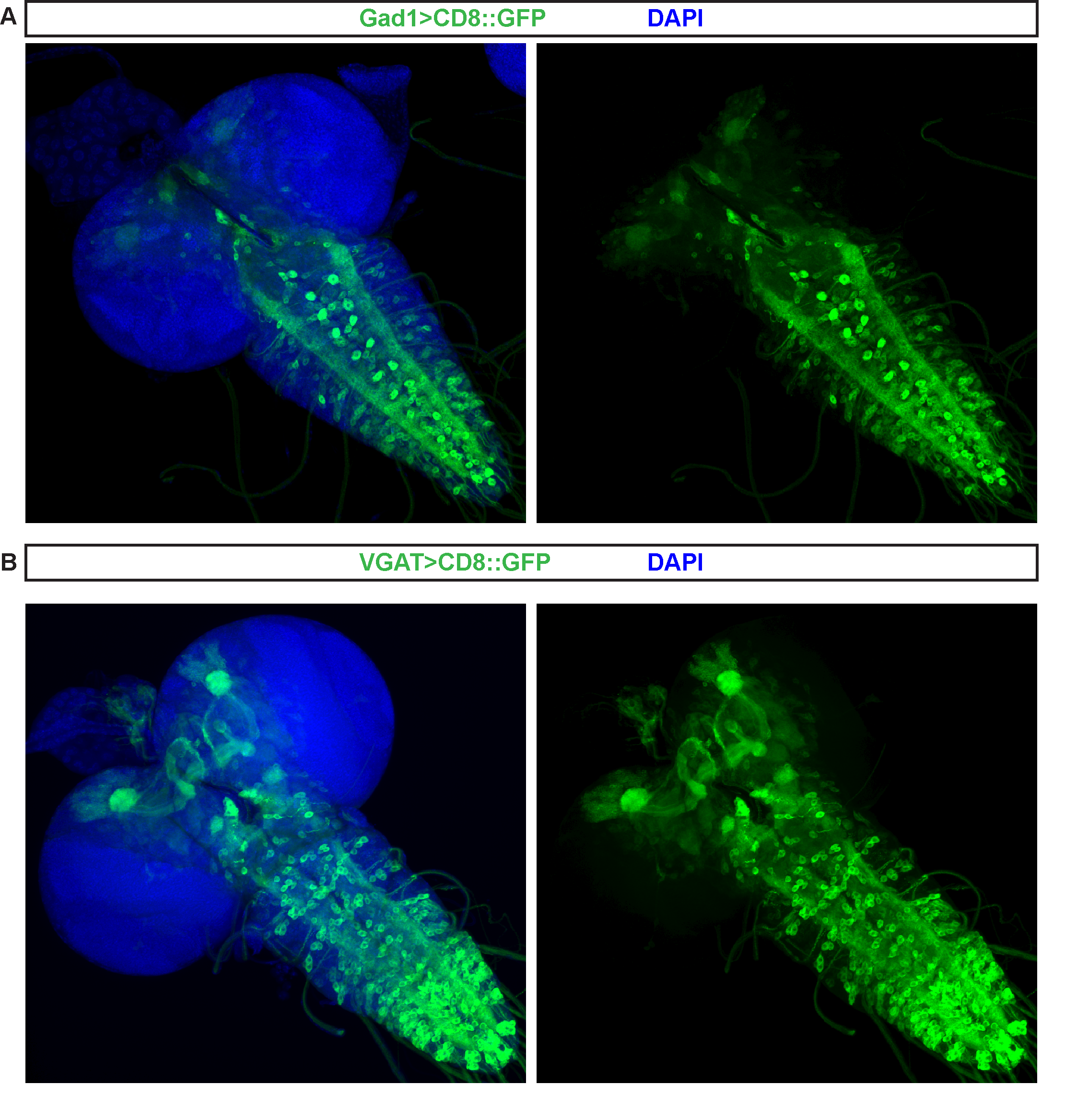

Supplement: S9 Fig — Confocal Z-projections of the CNSs from late L3 larvae showing Gad1-GAL4 (A) or VGAT-GAL4 (B) driving expression of membrane-targeted GFP (UAS-CD8::GFP) in green. DNA is stained by DAPI in blue. (TIF) [file pbio.2002252.s009.tif]

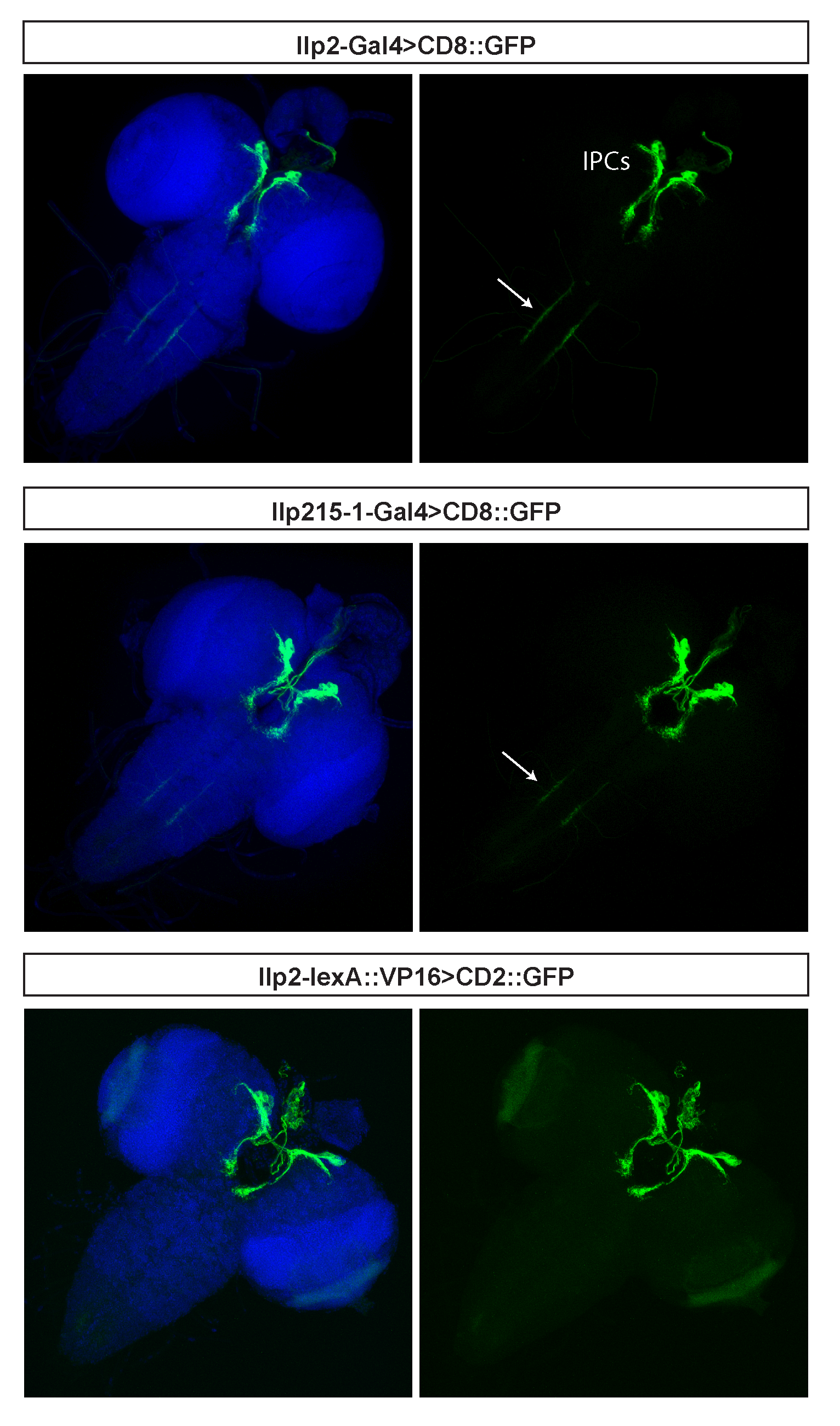

Supplement: S10 Fig — Confocal Z-projections of the CNSs from female late L3 larvae showing expression of membrane-targeted GFP (UAS-CD8::GFP or lexAOP-CD2::GFP) in green and DNA stained by DAPI in blue. Ilp2-Gal4 and Ilp215-1-Gal4 are weakly active in some neurons of the ventral nerve cord (VNC) and their projections (white arrows). Ilp2-lexA::VP16 shows no detectable expression in the VNC. (TIF) [file pbio.2002252.s010.tif]

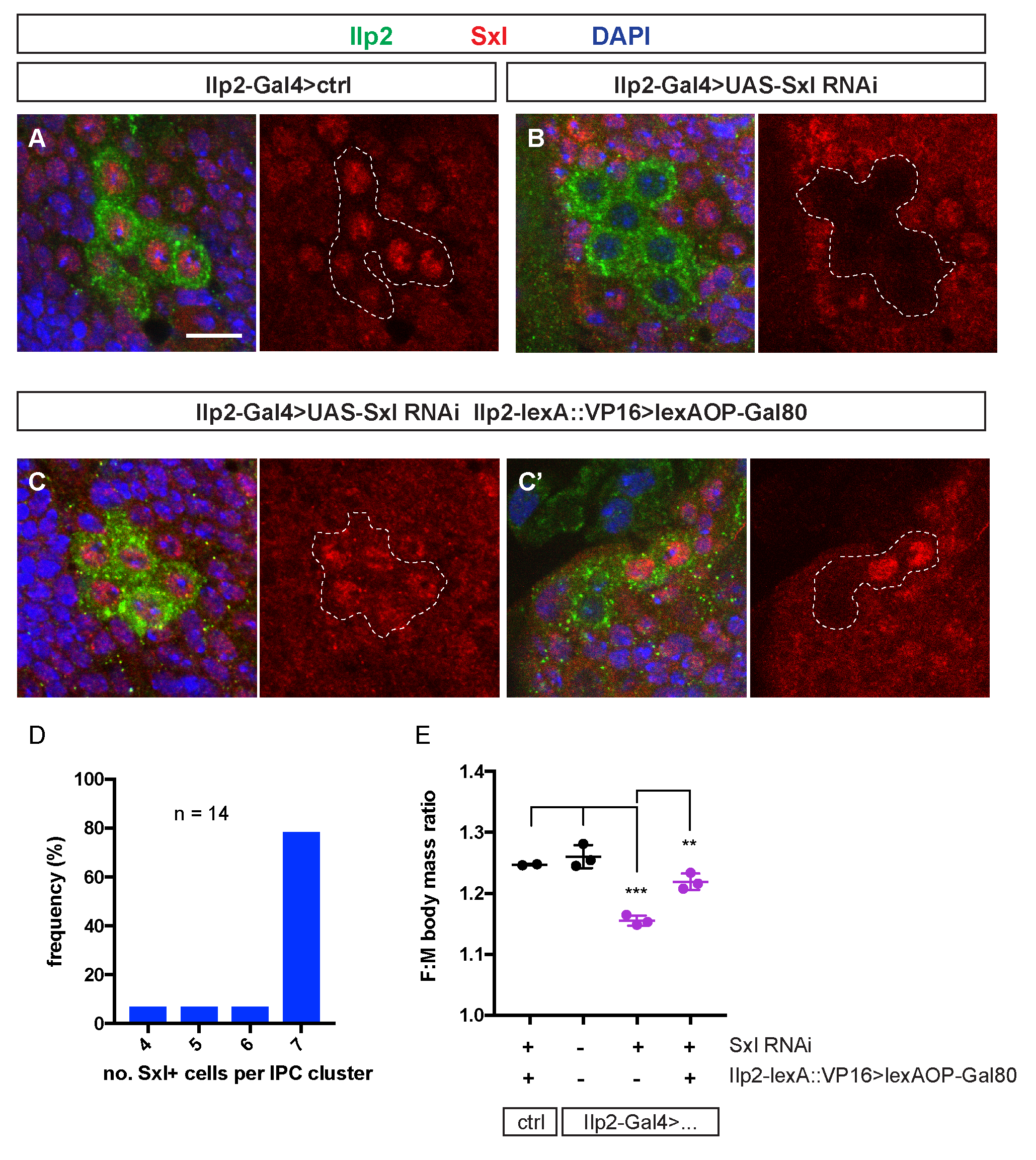

Supplement: S11 Fig — (A-C) Single confocal sections through clusters of IPCs from female larvae, immunostained for Sxl and Ilp2 (to mark IPCs), with DNA stained by DAPI (blue). Sxl in the nuclei of IPCs is strongly expressed in control females (A) but absent in those of Ilp2-Gal4>UAS-Sxl RNAi 1 larvae (B). Driving Gal80 (which blocks Gal4 activity) specifically in the IPCs of Ilp2-Gal4>Sxl RNAi 1 female larvae using the LexAOP system (Ilp2-lexA::VP16>lexAOP-Gal80) restores Sxl expression in the IPCs either fully (C) or partially (C’). Scale bar: 10mm. (D) Sxl expression is restored in all seven IPCs of ~80% of the female larvae carrying Ilp2-Gal4>UAS-Sxl RNAi + Ilp2-lexA::VP16>lexAOP-Gal80 (same genotype as C). (E) Restoration of Sxl expression in the IPCs of Ilp2>Sxl RNAi larvae (same genotype as in C and D) rescues the female to male (F:M) body mass ratio (SSD) of wandering L3 larvae. The underlying data for this figure can be found in S1 Data. (TIF) [file pbio.2002252.s011.tif]

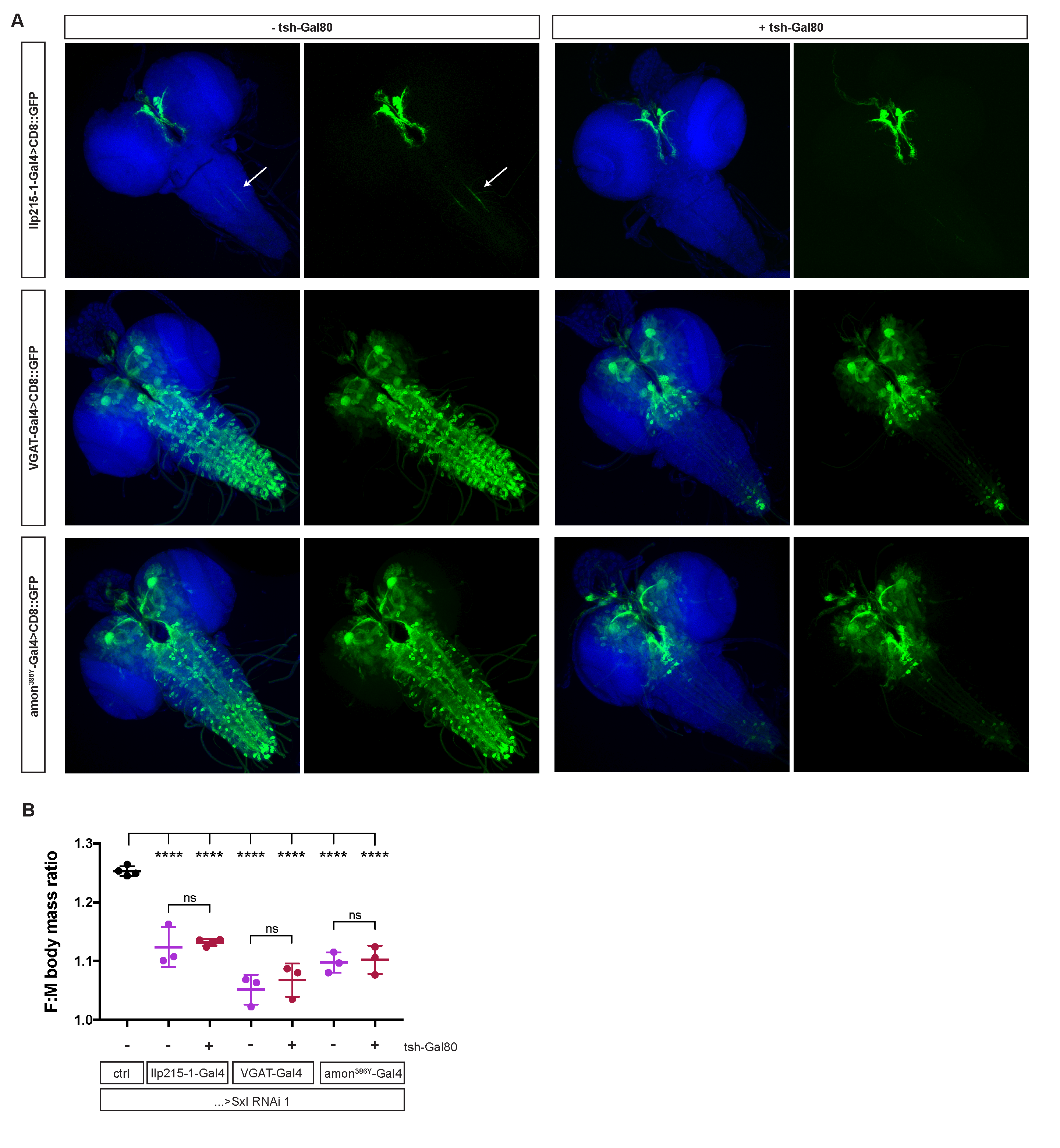

Supplement: S12 Fig — (A) CNS expression patterns in female larvae at late L3 of Ilp215-1-Gal4, VGAT-Gal4 or amon386Y-Gal4 driving UAS-CD8::GFP (green) in the absence (left) or presence (right) of tsh-Gal80. All images show confocal Z-projections with DNA stained by DAPI (blue). tsh-Gal80 efficiently supresses Gal4 activity in ventral nerve cord (VNC) neurons and their projections, including those in which Ilp215-1-Gal4 is expressed (white arrow and also see S9 Fig). (B) tsh-Gal80 does not suppress the decrease in larval body SSD resulting from Ilp215-1-Gal4, VGAT-Gal4 or amon386Y-Gal4 driving UAS-Sxl RNAi 1. Graph plots the mean and SEM of female to male (F:M) body mass ratios of wandering L3 larvae with individual data points representing mean F:M ratios from 3–4 independent experiments. **** indicates p<0.0001 and ns that p>0.05 using one-way ANOVA with multiple comparisons. The underlying data for this figure can be found in S1 Data. (TIF) [file pbio.2002252.s012.tif]

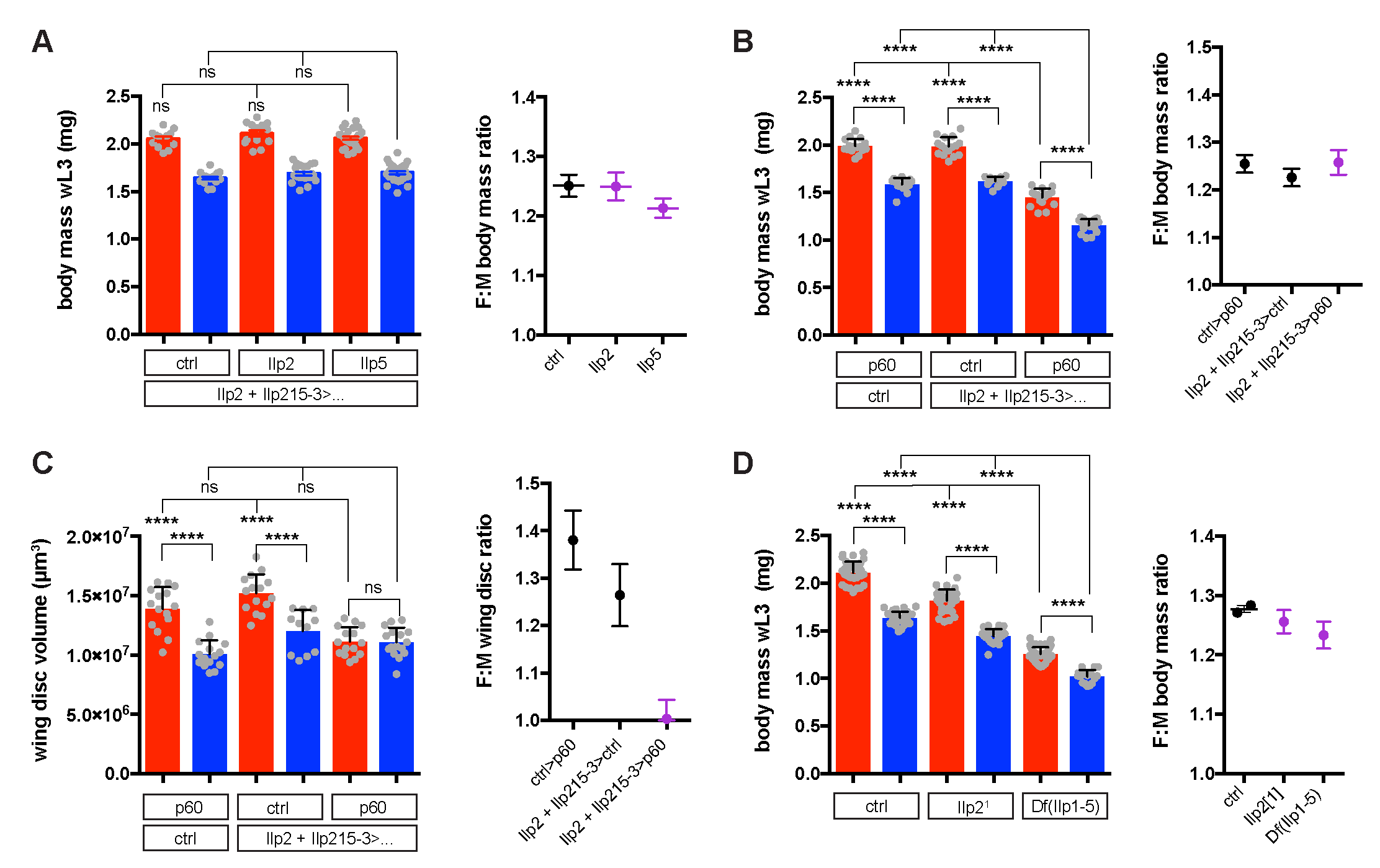

Supplement: S13 Fig — (A) IPC overexpression of UAS-Ilp2 or UAS-Ilp5 using a combination of Ilp2-GAL4 + Ilp215-3-Gal4 has no effect on body size or SSD. (B) IPC inhibition of PI3K signalling (UAS-p60 driven by Ilp2-GAL4 + Ilp215-3-Gal4) reduces body size in both males and females but it does not decrease larval body SSD. (C) IPC inhibition of PI3K signalling (UAS-p60 driven by Ilp2-GAL4 + Ilp215-3-Gal4) reduces the female size and SSD of wing imaginal discs. (D) Ilp2 null mutant (Ilp21) and Ilp1,2,3,4,5-deficient (Df(Ilp1-5)) larvae have decreased male and female body mass but no change in larval body SSD. Data for all graphs were obtained from wandering L3 larvae. Histograms show means, SD and individual data points. Mean female to male ratios (F:M ratios) and SEMs refer as indicated either to body mass or to wing disc volume. * indicates p<0.05 and **** indicates p<0.0001 using one-way ANOVA with multiple comparisons. The underlying data for this figure can be found in S1 Data. (TIF) [file pbio.2002252.s013.tif]

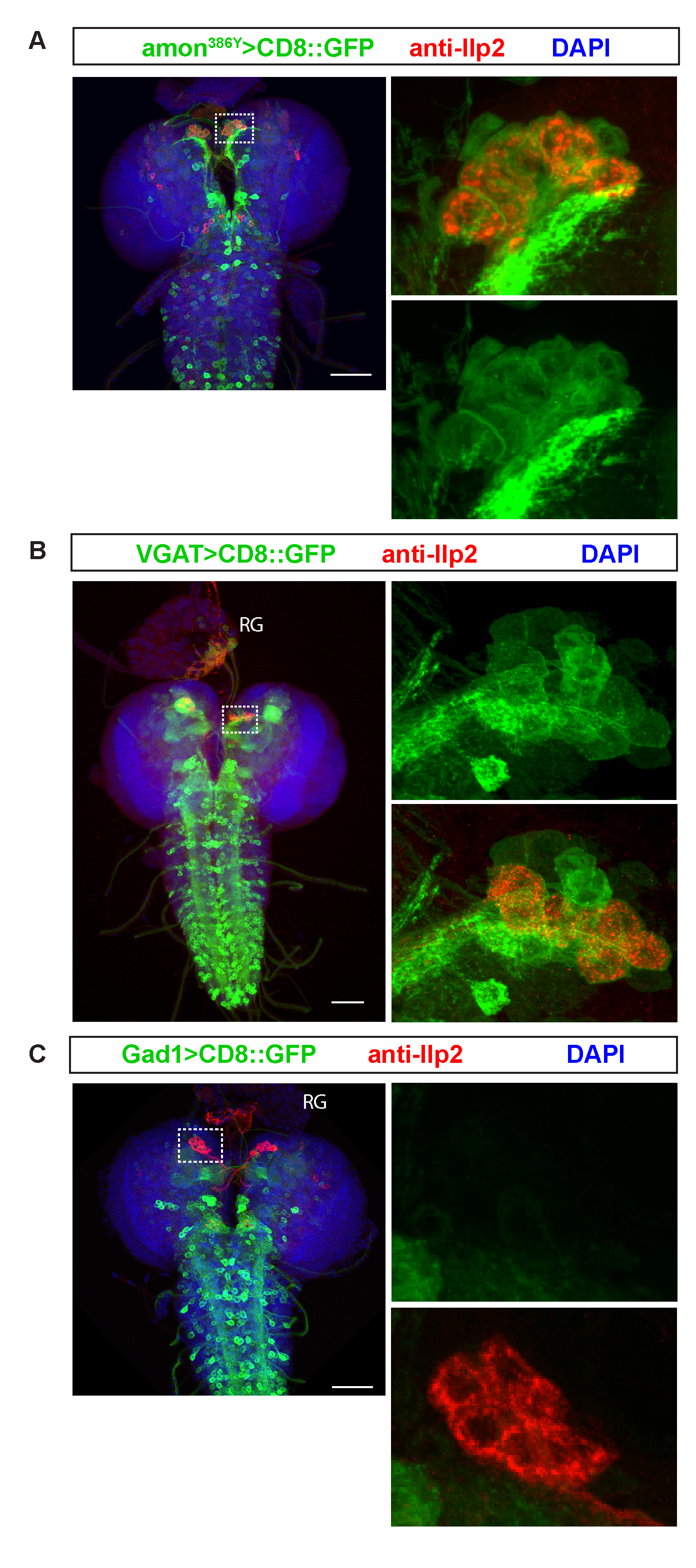

Supplement: S14 Fig — Confocal Z-projections of CNSs from late L3 larvae. UAS-CD8::GFP expression reveals that amon386Y-Gal4 (A) and VGAT-Gal4 (B) but not Gad1-Gal4 (C) are expressed in IPCs (marked by anti-Ilp2 immunostaining, Ilp2 also marks IPC projections in the corpora cardiaca of the ring gland, RG). Low power views of the CNS (left panels, scale bar 50mm) and higher magnifications of the IPCs (right panels, corresponding to boxed region in left panels) are shown. The signal intensity of GAL4 driver expression (green) has been increased in the right panels. (TIF) [file pbio.2002252.s014.tif]

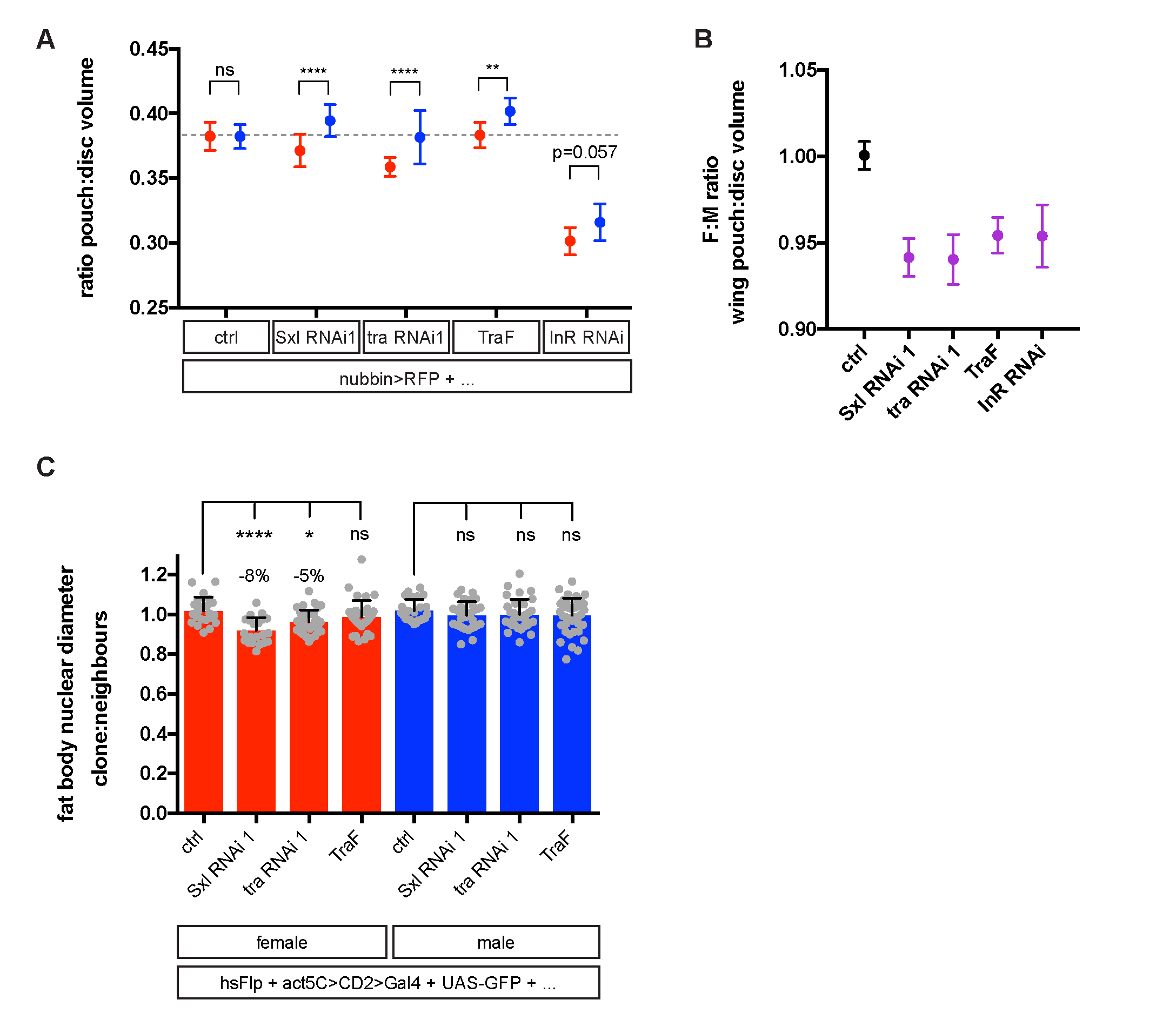

Supplement: S15 Fig — (A-B) nubbin-Gal4 driving expression of UAS-Sxl RNAi 1, UAS-tra RNAi 1, UAS-TraF or UAS-InR RNAi decreases the SSD of the wing pouch. Gal4-expressing cells in the wing pouch were marked by UAS-RFP. To measure cell-autonomous effects, the mean ratios and SEMs of the volumes of the wing pouch (expressing nubbin-Gal4) were normalised to the volumes of the whole wing disc (A), although normalization to only the non-pouch region of the disc gives similar results. Note that Sxl and Tra depletion specifically reduce female wing pouch volume, whereas TraF overexpression specifically increases male wing pouch volume. Insulin receptor (InR) knockdown reduces wing pouch sizes in both males and females but the effect is stronger in females. The corresponding mean and SEMs for female to male (F:M) ratios of wing pouch volumes normalised to whole wing disc volumes are also shown (B). Note that for this type of F:M ratio, control genotypes have a value of ~1.0 and that the values of less than 1.0 seen with all three genetic manipulations indicate decreased wing pouch SSD. (C) Decreased nuclear diameter of fat body cells expressing UAS-Sxl RNAi 1 or UAS-tra RNAi 1 but not UAS-TraF. Fat body clones were generated with hsFlp; actin5C>CD2>Gal4 and marked with UAS-nls::GFP. Graph plots the mean ratios of the nuclear diameter (a proxy for cell size) of GFP+/GFP- fat body cells. Each data point shows the mean ratio from one clone and error bars indicate the SD. Sxl and Tra knockdown decrease nuclear diameter in the fat body of females, whereas TraF overexpression has no effect on nuclear diameter in the fat body of either sex. The underlying data for this figure can be found in S1 Data. (TIF) [file pbio.2002252.s015.tif]

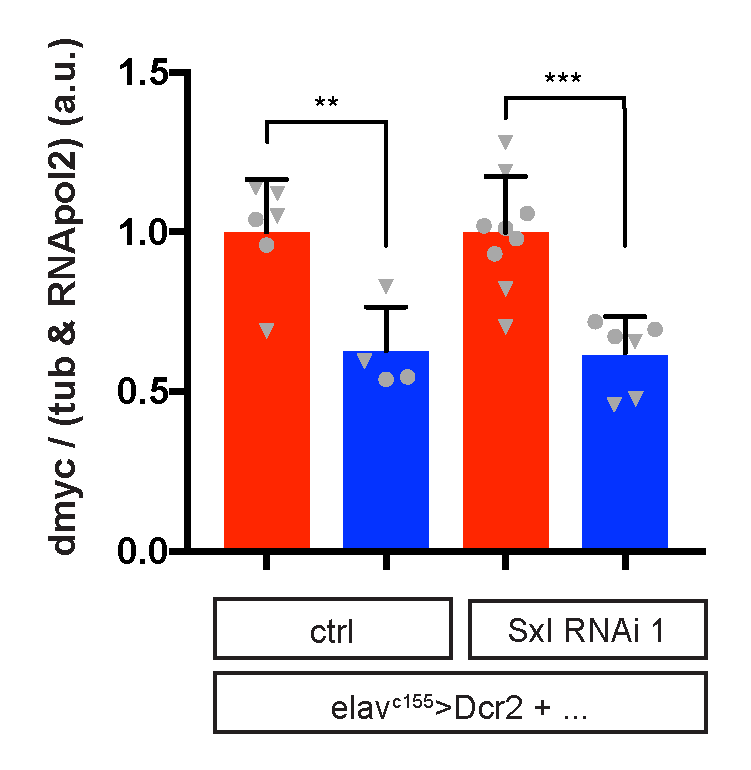

Supplement: S16 Fig — Graph shows qPCR quantitation of dmyc transcript levels from whole early L3 larvae, normalised to the geometric mean of tubulin and RNA polymerase II transcript levels. Individual data points are shown for replicates normalised to the mean female value for each genotype and pooled from two independent experiments (circles and triangles). Control larvae express dmyc at levels ~1.6 fold higher in females than males and this difference is retained in elavc155>Sxl RNAi larvae despite their loss of larval body SSD (see Fig 3A). ** indicates p<0.01 and *** shows p<0.001 using one-way ANOVA with multiple comparisons. The underlying data for this figure can be found in S1 Data. (TIF) [file pbio.2002252.s016.tif]
