# Supplementary material for: The sex of specific neurons controls female body growth in Drosophila
Source: PLoS Biol. 2017 Oct 4;15(10):e2002252. doi: 10.1371/journal.pbio.2002252 (PMC5627897; doi:10.1371/journal.pbio.2002252)
Supplement: S1 Table — Expression patterns were examined in late L3 larvae, with Gal4 drivers activating the expression of UAS-CD8::GFP. For elavc155-Gal4, expression was examined using act>stop>Gal4, UAS-GFP/GlaBc; UAS-Flp/TM6 to permanently label Gal4-expressing cells. An X or listed structures indicates expression present in some or all cells of this tissue/structure. Abbreviations: CC, corpora cardiaca; CNS, central nervous system; ECs, enterocytes; EEs, enteroendocrine cells; mNSCs, median neurosecretory cells; PNS, peripheral nervous system; SG, salivary gland; SNS, somatogastric nervous system; VNC, ventral nerve cord. “GABAergic” refers to the observation that expression of these Gal4 drivers only overlaps partially with GABA+ neurons in the early L3 female CNS (see S1 and S2 Images). (DOCX) [file pbio.2002252.s017.docx]

|  | **CNS** | **mNSCs** | **ring gland** | **SNS** | **hindgut nerve** | **EEs** | **ECs** | **inka cells** | **SG** | **other** |
| --- | --- | --- | --- | --- | --- | --- | --- | --- | --- | --- |
| **elav^c155^** | broad neuronal | x | CC | x | x | x | x | ? | x | some tracheal cells, all PNS, ovary |
| **dimm^c929^** | 309 peptiderigic neurons^1^ | x | CC | x |  | x |  | x | x | segmental lateral bipolar neurons of PNS^1^ |
| **amon^386Y^** | broad peptidergic | x | CC | x | x | x |  | x | x |  |
| **Ilp2** | IPCs and pairs of 3-4 neurons in VNC | IPCs |  |  |  |  |  |  | x | posterior spiracles |
| **Gad1** | broad “GABAergic” |  |  |  |  |  |  |  |  |  |
| **dVGAT** | broad “GABAergic” | x | CC? | x |  | x |  |  | x |  |

**S1 Table: Expression patterns of Gal4 drivers**

Expression patterns were examined in late L3 larvae, with Gal4 drivers activating the expression of *UAS-CD8::GFP*. For *elav^c155^-Gal4*, expression was examined using *act>stop>Gal4, UAS-GFP/GlaBc; UAS-Flp/TM6* to permanently label Gal4-expressing cells. An X or listed structures indicates expression present in some or all cells of this tissue/structure. Abbreviations: CC, corpora cardiaca; CNS, central nervous system; ECs, enterocytes; EEs, enteroendocrine cells; mNSCs, median neurosecretory cells; PNS, peripheral nervous system; SG, salivary gland; SNS, somatogastric nervous system; VNC, ventral nerve cord. “GABAergic” refers to the observation that expression of these Gal4 drivers only overlaps partially with GABA+ neurons in the early L3 female CNS (see S1 Image and S2 Image).

^1^ described in [1].

**References**

1. Park D, Veenstra JA, Park JH, Taghert PH. Mapping peptidergic cells in Drosophila: where DIMM fits in. PLoS One. 2008;3(3):e1896. doi: 10.1371/journal.pone.0001896. PubMed PMID: 18365028; PubMed Central PMCID: PMCPMC2266995.
